# Bifunctional Electrolyte Additive Enabling Simultaneous Interphase Formation on Both Electrodes in High‐Energy Lithium‐Ion Batteries

**DOI:** 10.1002/smll.202505772

**Published:** 2025-09-01

**Authors:** Ankita Das, Felix Pfeiffer, Anindityo Arifiadi, Matthias Weiling, Verena Küpers, Masoud Baghernejad, Martin Winter, Frank Glorius

**Affiliations:** ^1^ University of Münster Institute of Organic Chemistry Corrensstr. 36 48149 Münster Germany; ^2^ University of Münster, International Graduate School for Battery Chemistry, Characterization, Analysis Recycling and Application (BACCARA) Corrensstr. 40 48149 Münster Germany; ^3^ Helmholtz Institute Münster, IMD‐4 Forschungszentrum Jülich GmbH Corrensstr. 46 48149 Münster Germany; ^4^ University of Münster MEET Battery Research Center Corrensstr. 46 48149 Münster Germany

**Keywords:** bifunctional, cathode electrolyte interphase, electrolyte additives, high‐energy LIB, solid electrolyte interphase, thiophene, vinylene carbonate

## Abstract

The development of next‐generation Lithium‐ion batteries (LIBs) to meet the demands of advancing technology and energy storage requires focus on the formation of effective interphases on both the positive and negative electrodes. Different promising approaches to facilitate effective interphase formation are already known Out of these, the incorporation of film‐forming electrolyte additives is a straight‐forward strategy to achieve this goal. In the presented study, a bifunctional electrolyte additive, (5‐methyl‐2‐oxo‐1,3‐dioxol‐4‐yl)methyl thiophene‐3‐carboxylate composed of two functional motifs, vinylene carbonate (VC) and thiophene, is reported. Upon LIB operation, the additive undergoes in situ splitting, forming a VC‐assisted solid electrolyte interphase (SEI) and a polythiophene‐based cathode electrolyte interphase (CEI) simultaneously. The electrochemical performance of the proposed additive is studied in 250 mAh NMC811||AG + 20% SiO*_x_* wound pouch cells, and shows considerable improvement in overall battery performance compared to cells with the baseline electrolyte. The additive's dual interphase formation is confirmed through a combination of advanced characterization techniques, including X‐ray photoelectron spectroscopy (XPS), scanning electron microscopy (SEM), and  operando shell‐isolated nanoparticle‐enhanced Raman spectroscopy (SHINERS). This study introduces a new design strategy for a multifunctional electrolyte additive, providing a promising pathway to improve overall LIB's performance and lifetime by simultaneous stabilization of both electrodes through facilitated interphase formation.

## Graphical abstract

This article investigates a bifunctional electrolyte additive combining vinylene carbonate and thiophene to enhance high‐voltage lithium‐ion batteries. The additive enables simultaneous interphase formation on both electrodes. Electrochemical tests and advanced techniques such as XPS, operando‐SHINERS, and SEM reveal improved electrode stabilization and overall battery performance with this single‐additive strategy.

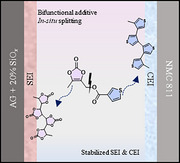

## 1 Introduction

LIBs have become a fundamental technology in the modern energy storage, acclaimed for its high‐energy density, specific capacity, and its versatility in applications ranging from consumer electronics, grid energy storage and electric vehicles (EVs).^[^ ^1^, ^2^, ^3^, ^4^ ^]^ Further improvements in lifespan, cost, and energy density are essential to meet the evolving requirements particularly the electric vehicle industry, as well as other sectors.^[^ ^5^, ^6^ ^]^ The use of Si‐based anode materials and Ni‐rich layered oxide cathode materials is an ubiquitous choice for EV application owing to the high energy storage capacities of these materials. However, employing both these systems together presents its own set of challenges.^[^ ^7^ ^]^

The potential of Si‐based anode material lies in its exceptionally high specific capacity of 3579 mAh g^−1^ (based on the fully lithiated phase Li_15_Si_4_), compared to 372 mAh g^−1^ for graphite (based on fully lithiated phase LiC_6_), representing nearly a ten‐fold increase. Nonetheless, the large volume expansions associated with lithiation of Si leads to particle cracking, loss of electronic contact and active material, resulting in severe capacity fade.^[^ ^8^ ^]^ To circumvent this challenge, typically Si is incorporated in smaller quantities (10–20 wt.%), in the form of SiO*_x_*,^[^ ^9^ ^]^ into graphite anodes.^[^ ^10^ ^]^ However, even in this configuration, the formation of an effective SEI is desirable to limit active Li‐loss due to continuous SEI breakage und subsequent reformation.^[^ ^11^ ^]^

Another key component in achieving high energy density is the incorporation of higher Ni contents in cathode in the form of Ni‐rich layered oxide materials.^[^ ^12^ ^]^ Energy density can be further elevated by increasing the upper cut‐off voltage that pushes the degree of cathode delithiation, providing an increased number of active lithium ions. On the contrary, the cathode potentials often exceed the oxidative stability of state‐of‐the‐art carbonate‐based electrolytes, thus leading to the formation of a resistive CEI.^[^ ^13^, ^14^ ^]^ Furthermore, a higher degree of cathode delithiation promotes transition metal (TM) dissolution through irreversible phase transitions from the cathode. These TM ions subsequently deposit on the anode side, poisoning the SEI and facilitate Li‐dendrite formation. The formation of Li‐dendrites results in the rapid loss of capacity, known as “sudden death” or “roll‐over” failure, and poses a severe safety hazard in extreme cases.^[^ ^15^, ^16^ ^]^

**Figure 1:**
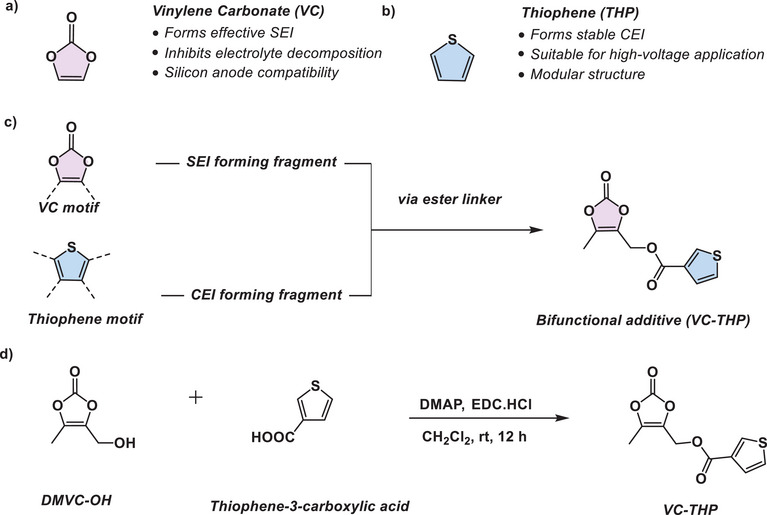
Schematic overview of the design principle of the VC‐THP additive comprising of a) VC, which serves as an SEI forming additive, and b) thiophene, which is employed to facilitate CEI formation. c) Presents the strategic incorporation of both active fragments resulting in the introduced VC‐THP additive. d) Synthetic route toward VC‐THP.

One promising solution to address the challenges associated with employing both Si‐based anode and Ni‐rich cathode is the incorporation of agents that facilitate the formation of a robust and effective SEI and CEI.^[^ ^17^, ^18^, ^19^ ^]^ Electrolyte additives are among the most common and effective way of facilitating interphase formation, greatly enhancing the stability and galvanostatic cycling performance of the LIBs.^[^ ^20^, ^21^, ^22^ ^]^ Numerous film‐forming additives for both anode and cathodes have already been reported.^[^ ^19^, ^23^, ^24^, ^25^, ^26^, ^27^, ^28^ ^]^ Fluoroethylene carbonate (FEC) is commonly used in LIBs with SiO*_x_*‐based anodes due to its ability to form a beneficial SEI.^[^ ^29^, ^30^, ^31^ ^]^ However, FEC is prone to defluorination reactions in the presence of Lewis acids that produce HF, increasing TM dissolution, which leads to the formation of various side products such as H_3_OPF_6_ and HPO_2_F_2_.^[^ ^32^, ^33^ ^]^ Vinylene carbonate (VC) is another widely utilized electrolyte additive specifically applied to form a mechanically durable SEI (**Figure** **1**a).^[^ ^34^, ^35^, ^36^ ^]^ It has been shown that VC‐derived SEIs consist of poly(VC) species, which form a flexible surface film, coping with the severe volume changes in Si‐based anodes.^[^ ^37^ ^]^ Despite the improved mechanical properties, VC‐derived interphases often struggle with increased Li ion resistance.^[^ ^38^, ^39^ ^]^

Recent research has increasingly focused on investigating CEI formation and identifying additives that can mitigate TM dissolution and prevent oxidative electrolyte decomposition. Several film‐forming additives specifically targeting the CEI, such as diphenyl diselenide^[^ ^40^ ^]^ and lithium difluoro(oxolato)borate,^[^ ^41^ ^]^ have been studied. Recently, thiophene‐based additives have also gained attention due to their ability to form conductive poly‐thiophene polymers, which protect the cathode and suppress further electrolyte decomposition (Figure 1b).^[^ ^42^, ^43^ ^]^

To simultaneously address the challenges of anode and cathode, the ideal solution would be to introduce a bifunctional agent. This molecule would undergo an in situ splitting reaction, releasing separate active fragments. One of these fragments promotes SEI formation on the anode, while the other fragment facilitates CEI formation on the cathode, effectively enhancing both interphases at the same time. Here, it is proposed that a carefully designed additive could combine the advantages of VC and thiophene, offering improved interphase properties (Figure 1c).

Herein, the design and synthesis of a bifunctional electrolyte additive derived from VC and thiophene core motifs is presented. These two motifs are linked through a carboxylic ester group, resulting in the additive (5‐methyl‐2‐oxo‐1,3‐dioxol‐4‐yl)methyl thiophene‐3‐carboxylate (VC‐THP) (Figure 1d). The additives’ influence on the galvanostatic cycling performance is investigated in 250 mAh multilayer pouch cells with a NMC811||AG + 20% SiO_x_. At an optimized concentration, VC‐THP notably enhances the electrochemical performance and long‐term cycling stability of the cells. To clarify the initial hypothesis that the additive undergoes in situ splitting to form simultaneous interphases on both electrodes, a wide range of complementary techniques were employed, including SHINERS, XPS, SEM, and preliminary density functional theory (DFT) calculations.

## 2 Results and Discussion

### 2.1 Electrochemical Results

**Figure 2:**
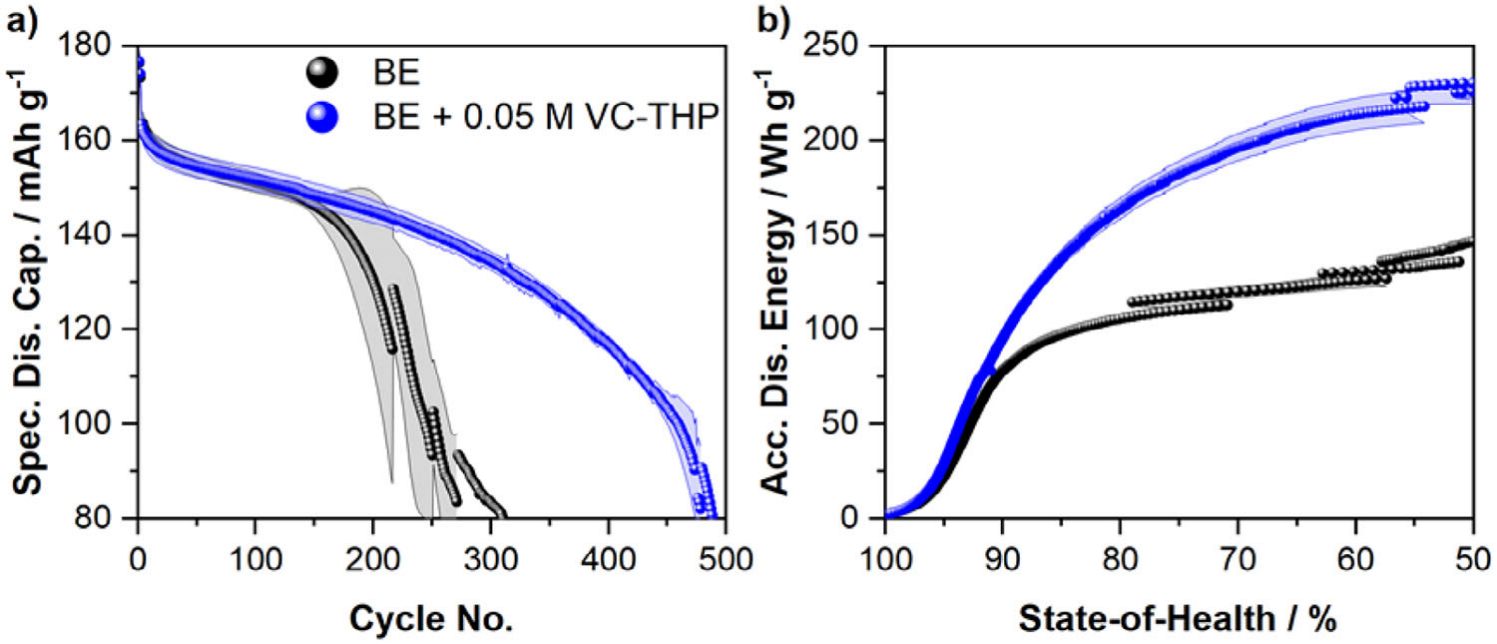
Results of the galvanostatic cycling experiments of NMC811||AG + 20% SiO_x_ pouch cells. The cells were cycled in the presence of the baseline electrolyte (BE, 1 m LiPF_6_ in EC:EMC, 3:7 by weight; black) and the BE + 0.05 m VC‐THP (blue). Shown are a) the specific discharge capacities and b) the accumulated specific discharge energies.

The influence of the proposed electrolyte additive, VC‐THP on the performance of high‐voltage NMC811||AG + 20% SiO_x_ multilayer pouch cells was investigated via galvanostatic charge/discharge cycling experiments. The results of the performed investigations are depicted in **Figure** **2**. Note that the presented figures only contain the data for the optimized electrolyte additive concentrations. Concentration optimization for the considered additive is shown in Figure S1 (Supporting Information).

In comparison to the BE cells, the VC‐THP‐containing cells exhibit a notably improved galvanostatic cycling performance (Figure 2a). Note that all given specific capacities are calculated based on the cathode active material. For the BE cells, a specific discharge capacity of 163 mAh g^−1^ was recorded after formation, decreasing to a value of 147 mAh g^−1^ after 150 charge/discharge cycles. For higher cycle numbers, the BE‐containing cells exhibit a severe capacity fading, concluding in an end‐of‐cycle life (50% state‐of‐health (SOH)) after ≈250 charge/discharge cycles. For the cells cycled in the presence of an optimized VC‐THP concentration, a similar specific discharge capacity of 163 mAh g^−1^ was obtained after formation. Moreover, the additive‐containing cells demonstrate a similar capacity fading, resulting in a capacity of 149 mAh g^−1^ after 150 charge/discharge cycles. However, in the presence of VC‐THP, the rapid capacity fading, observed for the BE cells, did not occur. Instead, the cells exhibit a cycle life of almost 500 charge/discharge cycles, doubling the cycle life of the BE cells. This also reflects on the accumulated specific discharge energies (ADEs), depicted in Figure 2b. Comparing the ADEs of cells cycled in the presence of different electrolyte formulations is a straightforward tool to evaluate the respective electrochemical performance comprehensively.^[^ ^42^, ^43^, ^44^ ^]^

Similar to the obtained discharge capacities, BE and BE + 0.05 m VC‐THP‐containing cells exhibit comparable ADE values for low cycle numbers. This changes notably after surpassing 90% SoH, where BE cells show only a minor increase in ADE values, due to the severe capacity fading. In contrast, VC‐THP‐containing cells reached an ADE value of 230 Wh g^−1^ at a SoH of 50%, greatly outperforming the BE cells, exhibiting an ADE value of 135 Wh g^−1^. Even at the commercially relevant mark of 80% SoH, VC‐THP cells show an ADE increase of ≈50%.

The rapid capacity fading observed for the BE cells can be attributed to the degradation of both anode and cathode active material. While anode degradation is mostly fueled by the mechanical stress during lithiation and de‐lithiation of the SiO*_x_* particles, cathode degradation is driven by the high‐voltage conditions during galvanostatic cycling. The high operational voltage sparks irreversible phase transition within the NMC bulk material, as well as the dissolution of transition metal ions into the electrolyte, resulting in the so called “roll‐over failure”.^[^ ^15^, ^16^, ^43^ ^]^ The implementation of effective interphases on both anode and cathode is a promising approach to cope both mentioned challenges. As discussed in detail in the introduction, this work is focused on a bifunctional electrolyte additive, facilitating both SEI and CEI formation. While the results of the galvanostatic cycling experiments already indicate the formation of beneficial interphase(s), further investigation was conducted to confirm interphase formation. The Coulombic efficiencies (CEs) depicted in Figure S2 (Supporting Information) show notably lower values during formation for the additive‐containing cells. For the first formation cycle, a CE of 78% was recorded in the presence of VC‐THP, compared to a CE of 78.3% observed for the BE cells. Interestingly, the difference increased for the second formation cycle, showing CEs of 96.6% and 97.2% for additive‐ and BE‐containing cells, respectively. During continuous charge/discharge cycling, similar CEs of 99.8% were observed for both considered electrolyte formulations. It is worth noting that toward the end of cycling, the CEs of the BE‐ and additive‐containing cells greatly decrease, mimicking the increased capacity fading. The obtained CEs clearly indicate the formation of an additional interphase in the presence of VC‐THP, accounting for the increased charge consumption. Moreover, the increasing CE difference in the second formation cycle could be attributed to a two‐phased interphase formation.

**Figure 3:**
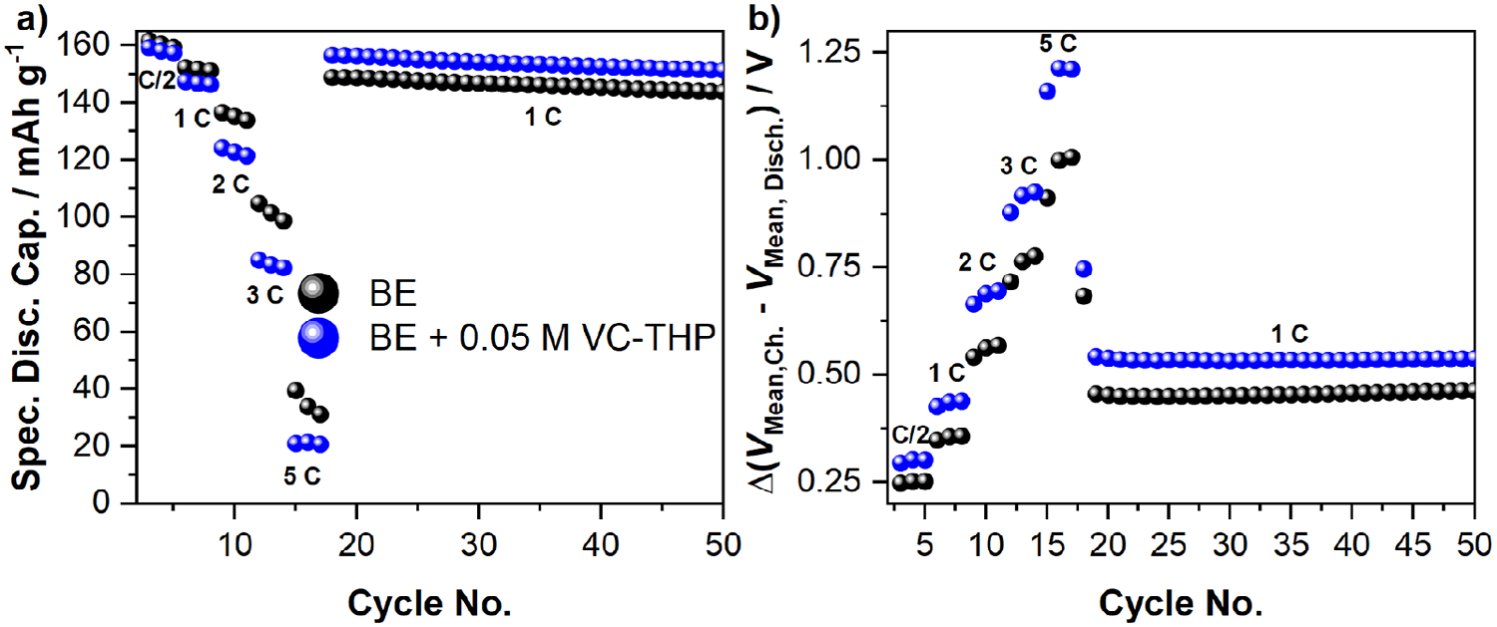
Results of the galvanostatic C‐rate investigation of NMC811||AG + 20% SiO_x_ pouch cells. The cells were cycled in the presence of the baseline electrolyte (BE, 1 m LiPF_6_ in EC:EMC, 3:7 by weight; **black**) and the BE + 0.05 m VC‐THP (**blue**). Shown are a) the obtained specific discharge capacities and b) the difference between the mean charge and mean discharge voltage (ΔV). The respective C‐rates are noted in the figures.

Further investigation on the electrochemical properties of the additive‐derived interphase(s) was conducted using a C‐rate investigation, depicted in **Figure** **3** below.

The specific discharge capacities recorded during the C‐rate investigations (Figure 3a), demonstrate notable differences for the cells containing the considered electrolyte formulations. While similar capacities were obtained for C/2 and 1 C, cells containing VC‐THP exhibited lower values for higher C‐rates. These findings are in good agreement with the mean voltage difference analysis (Δ*V*), shown in Figure 3b. The analysis of Δ*V* values is a straightforward tool to estimate the internal resistance of cells, providing basic insights, e.g., into interphase formation or electrolyte consumption during long term cycling.^[^ ^42^, ^43^, ^44^ ^]^ It is evident that the cell containing VC‐THP exhibits notably higher Δ*V* values across all applied C‐rates. The difference becomes especially apparent for 3 and 5 C. The higher Δ*V* values observed in the presence of the additive indicate the formation of VC‐THP‐derived interphase(s). This, in turn, increases internal resistance, leading to higher over‐voltages and, consequently reduced discharge capacities. Similar to the results shown in Figure 2a, for the continuous galvanostatic charge/discharge cycling conducted after the C‐rate investigation, higher discharge capacities were obtained for the VC‐THP containing cells, despite higher Δ*V* values. This could be attributed to a protective behavior of the formed interphase(s), suppressing electrode material degradation. However, also the lower rate of delithiation, due to the higher over‐voltages of the additive‐containing cells, has to be considered.

Despite the promising results obtained from galvanostatic charge/discharge cycling experiments, it remains unclear whether the presence of VC‐THP results only in the formation of only an SEI, only a CEI or both interphases. Additional galvanostatic cycling experiments, performed in the presence of an optimized VC concentration, provide initial insights into the raised question. The results shown in Figures S3 and S4 (Supporting Information) show slightly higher specific discharge capacities for the VC‐THP containing cells compared to those with VC alone. This trend is also reflected in the ADE, showing higher values for the VC‐THP cells, especially before 80% SoH, indicating a beneficial influence of the additive on the cathode side as well, resulting in enhanced performance. Nevertheless, the improved cycling performance, resulting in higher discharge capacity values, can also be held accountable for the higher rate of capacity fading at higher cycle numbers (below 80% SoH), observed for the VC‐THP cells. Due to the higher degree of (de‐)lithiation of the anode and cathode, additional mechanical stress is forced upon the electrode active materials, accelerating their respective degradation.

**Figure 4:**
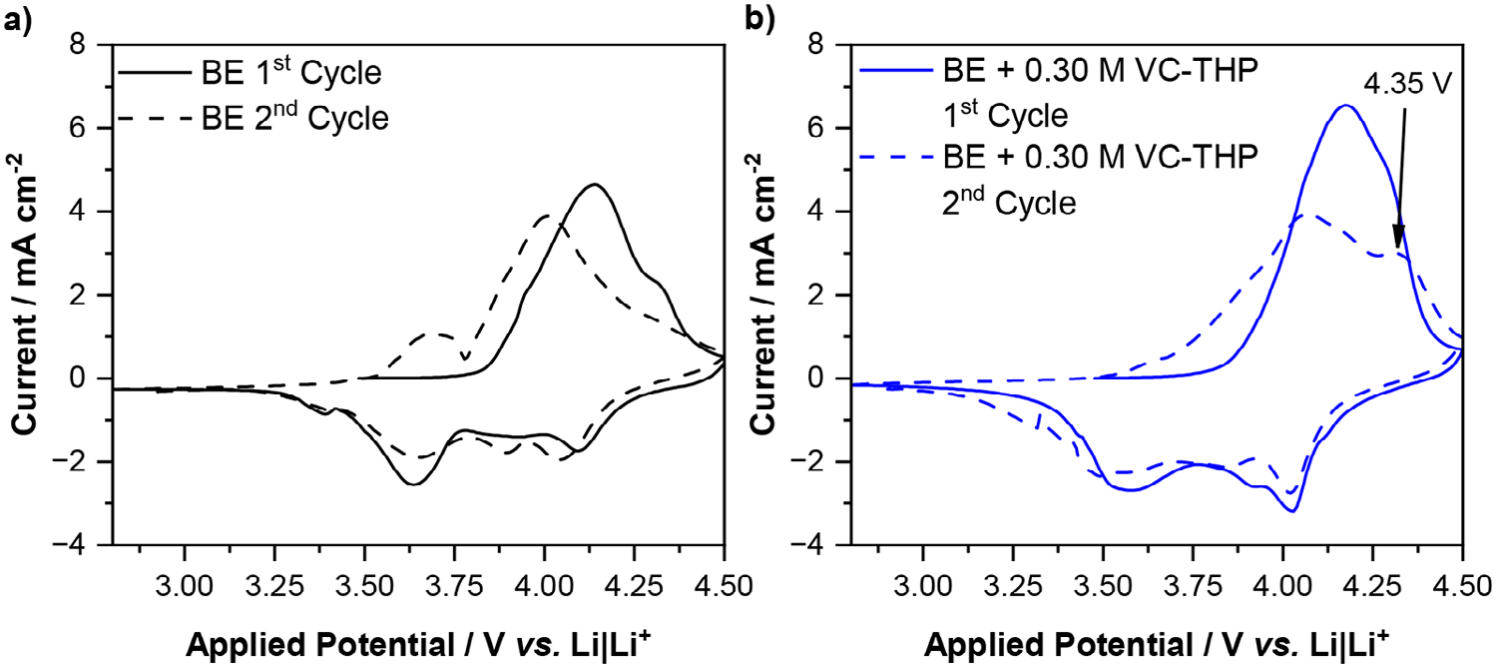
Cyclic voltammograms of NMC811||AG + 20% SiO_x_ T‐type Swagelok cells with Li‐metal reference electrodes performed in the presence of a) the baseline electrolyte (BE, 1 m LiPF_6_ in EC:EMC, 3:7 by weight; **black**) and b) the BE + 0.30 m VC‐THP (**blue**). Shown are the first and second CV cycles indicated by full and dashed lines. Prominent features are highlighted in the figure by an arrow, and the respective applied potential is noted.

While additional galvanostatic cycling experiments suggest the formation of a CEI in the presence of VC‐THP, potentiodynamic investigations offer further insights. **Figure** **4** presents cyclic voltammograms (CVs) obtained from NMC811|| AG + 20% SiO_x_ T‐type Swagelok cells with Li‐metal reference electrodes recorded in the presence of the BE and the BE + VC‐THP, representing electrochemical processes on the cathode.

The CVs of both considered electrolyte formulations are clearly dominated by features ascribed to the (de‐)lithiation of the NMC811 active material. However, in the second anodic cycle the CV of the additive‐containing electrolyte displays an additional prominent band at 4.35 V versus Li|Li^+^. Supposedly, this newly arising peak could be attributed to the polymerization of the thiophene motif on the surface of the cathode, resulting in the formation of an additive‐derived CEI, accounting for the improved galvanostatic cycling performance. In addition, the recorded potential of the suggested thiophene polymerization is in very good agreement with a previous study on thiophene as CEI‐forming additive.^[^ ^45^ ^]^ Furthermore, since the majority of thiophene polymerization occurs during the second cycle, this suggests that the VC‐THP additive undergoes a splitting reaction during the first cycle. In the CVs recorded from AG + 20% SiO_x_||Li‐metal T‐type Swagelok cells with Li‐metal reference electrodes, monitoring processes on the anode side, an additional peak was observed at 1.43 V versus Li|Li^+^ within the first cycle (Figure S6, Supporting Information). This peak corresponds to the reduction of the additive molecule, possibly including the splitting of the VC and thiophene motif, thus allowing thiophene polymerization in the subsequent cycle. Moreover, this suggestion is in good agreement with the observed CE values, showing an increased CE difference between BE and BE + 0.05 m VC‐THP cells from the first to the second cycle, possibly accounting for additional additive consumption within the second cycle. It is also worth noting that the reduction potential of the VC‐THP molecule is notably higher, compared to the single VC molecule, exhibiting reductive decomposition at a potential of 1.05 V versus Li|Li^+^ in a similar experimental set‐up.^[^ ^42^ ^]^

### 2.2 SEM Analysis of the Electrolyte−Electrode Interphases

**Figure 5:**
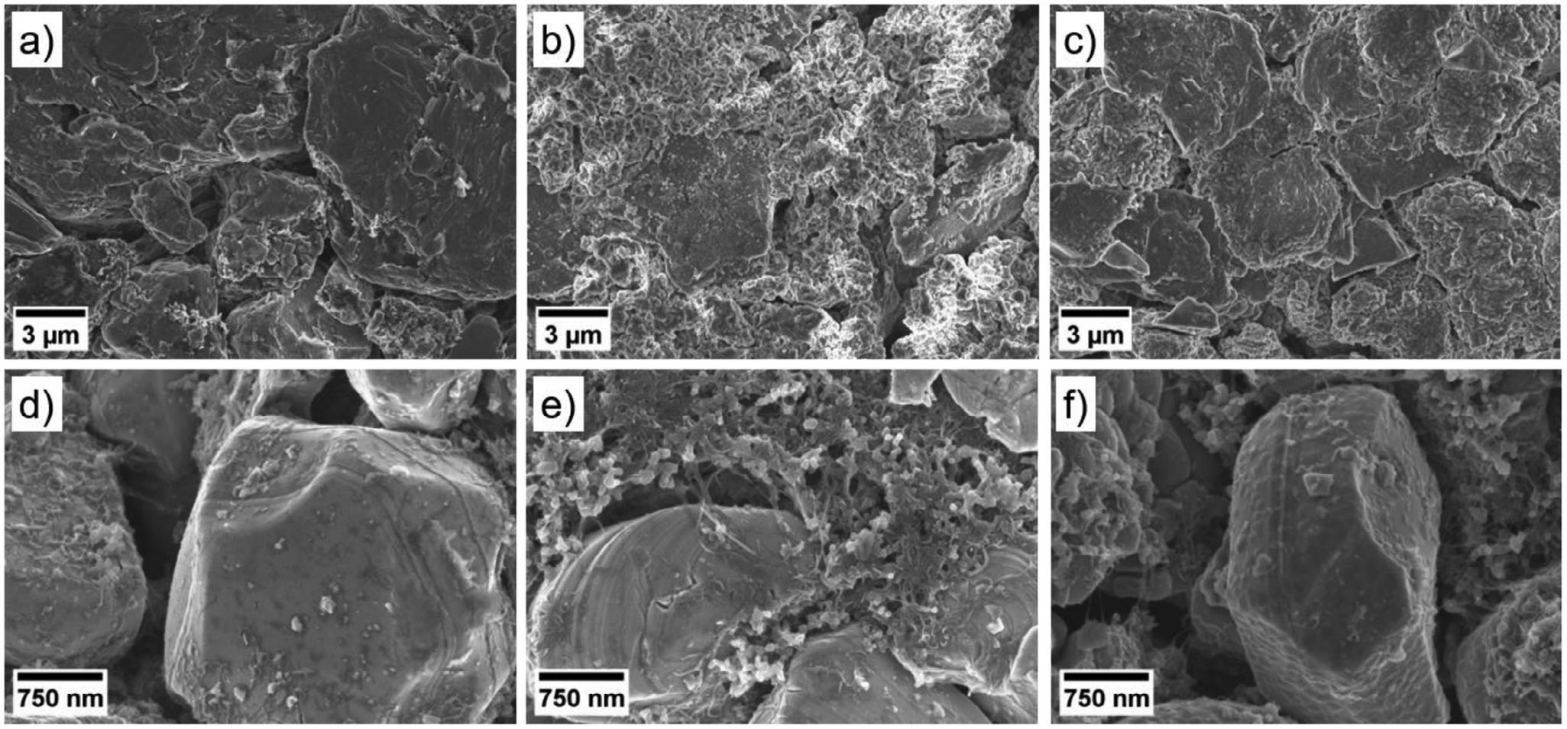
SEM images obtained from electrochemically aged electrodes taken from NMC811||AG + 20% SiO_x_ pouch cells after two formation cycles. SEM images of the anode are shown in figures (a–c), and images of the cathode are depicted in figures (d–f). The electrodes were electrochemically aged in the presence of a) + d) the baseline electrolyte (BE, 1 m LiPF_6_ in EC:EMC, 3:7 by weight), b) + e) the BE + 0.15 m VC, and c) + f) the BE + 0.05 m VC‐THP.

SEM analysis was conducted to visualize the interphases formed on both anode and cathode surfaces in the presence of VC‐THP. **Figure** **5** presents the SEM images of both electrodes extracted after two formation cycles from NMC811||AG + 20% SiO_x_ pouch cells cycled in the presence of the BE, the BE + VC, and the BE + VC‐THP. Based on the suggestion that the introduced VC‐THP additive splits during battery operation, electrodes electrochemically aged in the presence of VC were considered for the presented comparison.

For the anode surface, the distinct formation of a SEI was observed for all considered electrolyte formulations, described in detail in the following. Interestingly, the morphology of the different interphases strongly varied, depending on the respective electrolyte.

For the BE sample (a), the formation of a smooth layer was observed on the surface. In contrast, a rough SEI was formed in the presence of VC (b). In addition, the VC‐containing cells exhibited prominent Li plating on the anodes surface, showing a mossy structure on top of graphite and SiO*_x_* particles. Note that the cauliflower‐like structures in the shown SEM image are SiO*_x_* particles, while the graphite particles can be identified by their layered structure. The observed Li plating might cause the decreased discharge capacities monitored during the galvanostatic cycling experiments in the presence of VC (Figures S3 and S4, Supporting Information). For the VC‐THP sample (c) no prominent Li plating was observed. Instead, the formed SEI exhibited a rough morphology, similar as observed for VC. The similarity in the morphologies between the VC and the VC‐THP derived SEI was expected, as only the VC motif of the VC‐THP additive is intended to participate in the formation of the SEI. On the cathode side however, notable differences were captured. While the NMC811 electrodes extracted from the BE and the VC containing cells did not exhibit prominent CEI formation, a notable, scale‐like CEI was formed on the NMC particles in the presence of VC‐THP (Figure 5f).

These findings further support our hypothesis that the VC‐THP molecule splits during reduction, enabling the formation of an VC‐derived SEI and a thiophene‐derived CEI, thereby acting as a bifunctional additive.

### 2.3 X‐Ray Photoelectron Analysis of Electrodes

**Figure 6:**
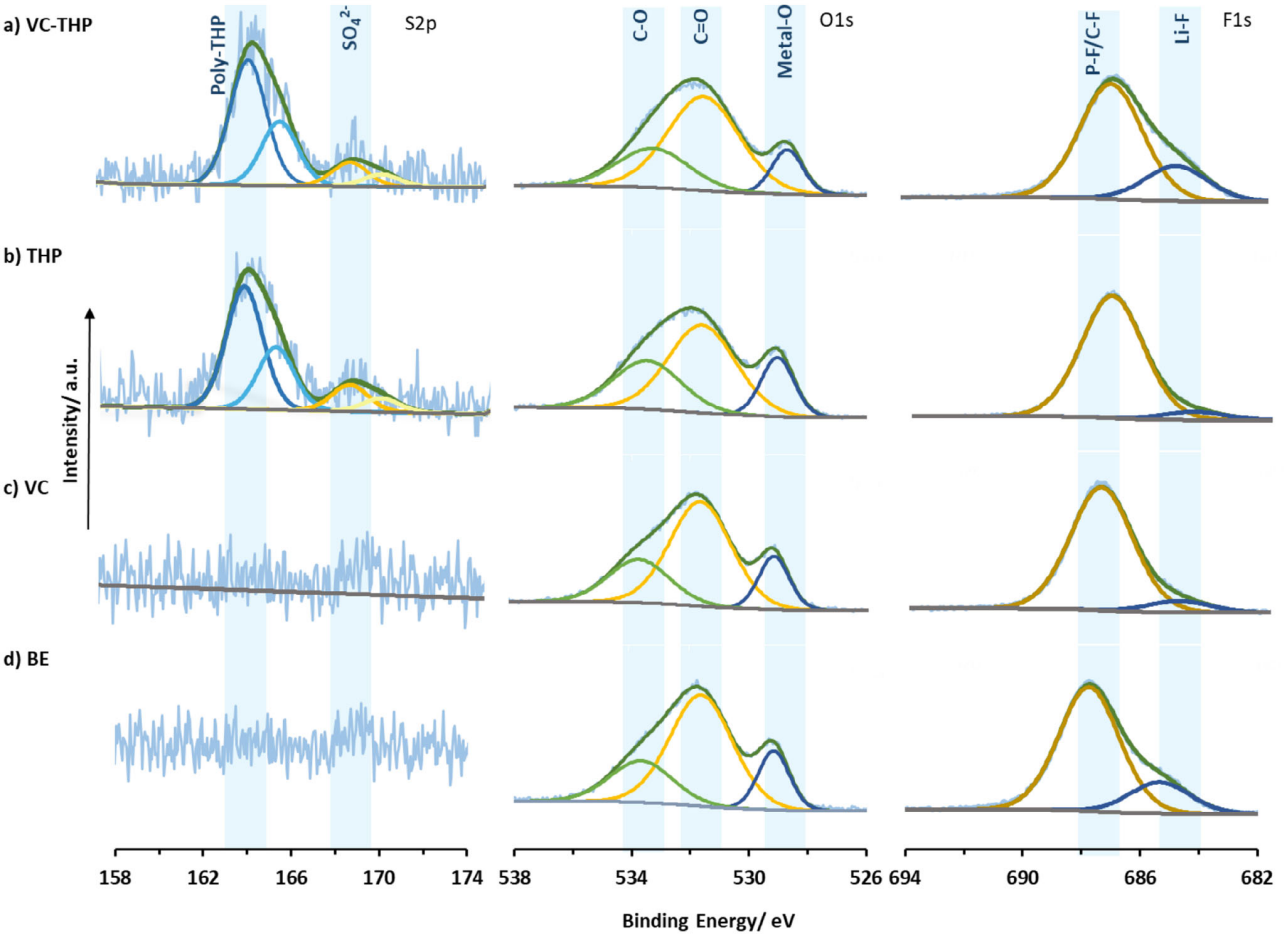
S2p, O1s, and F1s of NMC811 surface after formation cycles obtained via XPS. a) VC‐THP, b) thiophene (THP), c) VC, d) baseline electrolyte (BE, 1 m LiPF_6_ in EC:EMC, 3:7 by weight).

XPS analysis was conducted to investigate the SEI and CEI composition of the SiO_x_‐graphite anode and NMC811 cathode extracted after formation cycles, respectively. Here, the XPS data from four different cases, a) BE + VC‐THP, b) BE + thiophene only (THP), c) BE + VC only, and d) BE only (**Figure** **6**) are compared. On the cathode, a distinct sulfur doublet peak (S2p_3/2_, ≈164.1 eV) characteristic of polythiophene‐species^[^ ^46^, ^47^ ^]^ is observed in cells containing thiophene and VC‐THP as additives. This finding confirms that the thiophene motifs in these additives actively participates in constructing the CEI, as hypothesized. Additionally, a sulfate group peak at 168.3 eV is present along with polythiophene signal, likely resulting from the oxidation process. In case of O1s spectra, the metal‐O peak at ≈528.7 eV is present significantly in all cases, suggesting formation of thin CEI layers, among all cases, the cells containing only THP has the thinnest CEI (Table S3, Supporting Information). We also see higher Li−F (≈684.9 eV) contents in the CEI of the THP‐VC compared to using only VC and THP, indicating more LiPF_6_ decomposition (Table S4, Supporting Information).

**Figure 7:**
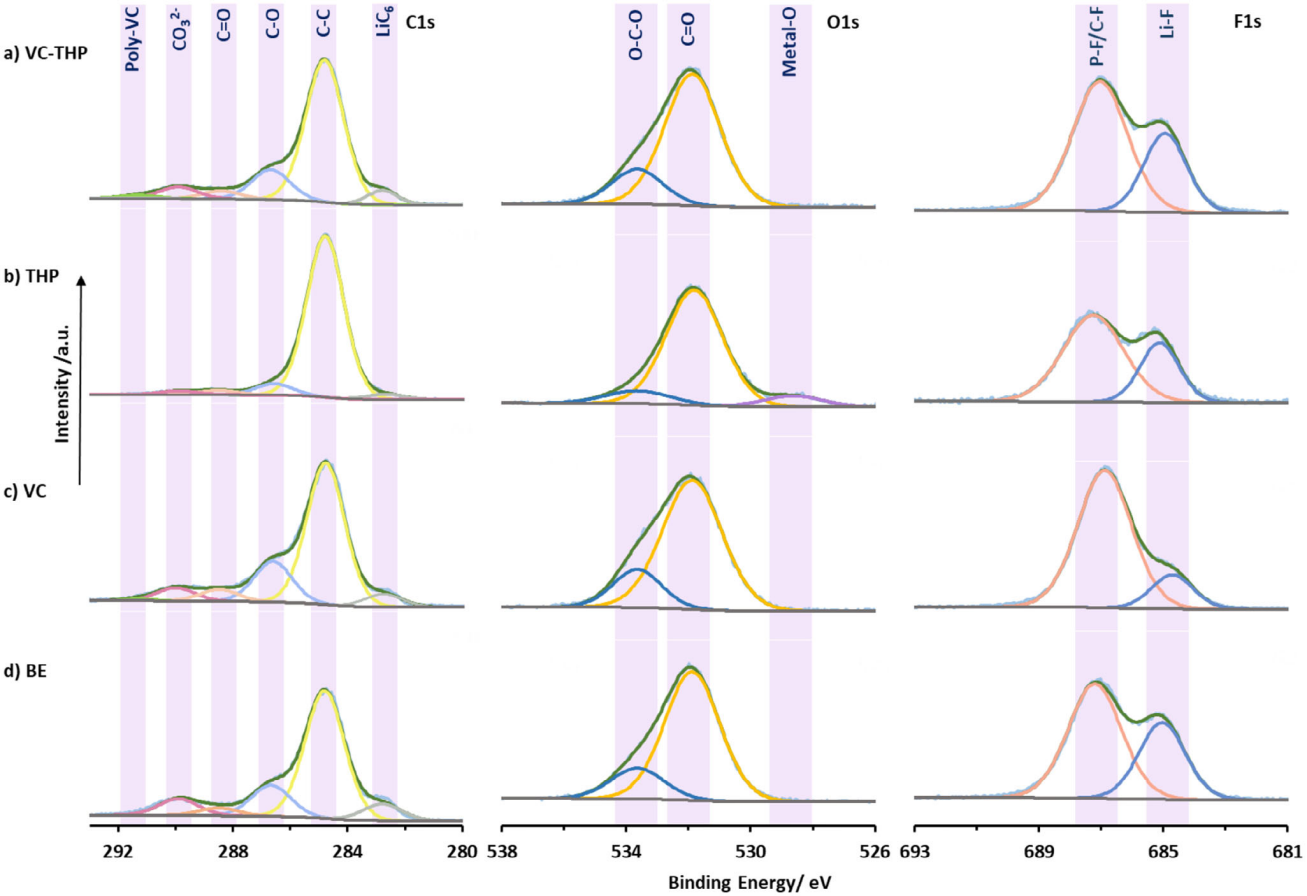
C1s, O1s, and F1s of SiO*_x_*‐graphite surface after formation cycles obtained via XPS. a) VC‐THP, b) thiophene (THP), c) VC, d) baseline electrolyte (BE, 1 m LiPF_6_ in EC:EMC, 3:7 by weight).

On the anode (**Figure** **7**), C1s spectra obtained from only VC containing cell and VC‐THP containing cells are very similar, with a notable peak at ≈291.3 eV in both cases, which can be related to the presence of poly‐VC species in the SEI (Table S5, Supporting Information).^[^ ^39^ ^]^ In contrast, a clear difference between the C1s spectra of the thiophene only and VC‐THP containing cells can be noted, the peaks associated with C─O, C═O, CO_3_^2−^ are more pronounced in case of VC‐THP suggesting participating of VC‐fragment along with other solvent decomposition products in the SEI formation. The O1s spectra for the SEI primarily consists of two peaks at ≈531.8 eV (C═O) and ≈533.7 eV (C─O). The ratios of these peaks are similar in the case of VC‐THP and VC, suggesting similarities in their SEI composition, in contrast to the case of THP (Table S6, Supporting Information). F1s spectra further reveals the differences in SEI components across four cases studied here. Two distinct peaks are observed, associated with P−F/C−F at ≈687.2 eV and its degradation product, Li−F at ≈684.9 eV. Interestingly, the VC‐containing cells exhibit the lowest LiF content and a slight shift of the P–F/C–F peak to 686.89 eV, which likely reflects a greater contribution from P–F species (e.g., Li_x_PO_y_F_z_) formed via LiPF_6_ decomposition. While the BE and VC‐THP cells had the highest Li−F contents. This observation suggest that VC most effectively suppresses LiPF_6_ degradation followed by the other cases (Table S7, Supporting Information).

A combined study of the SEI and CEI through XPS supports the in situ splitting of the VC‐THP additive, leading to the simultaneous formation of interphases on both the cathode and anode. Notably, no sulfur species were observed on the anode side for THP and VC‐THP, providing further evidence for the additive's splitting behavior enabling the specific reactions which results in the formation of additive derived interphases on both anode and cathode.

### 2.4 *Operando* SHINERS of the Cathode Electrolyte Interphase

**Figure 8:**
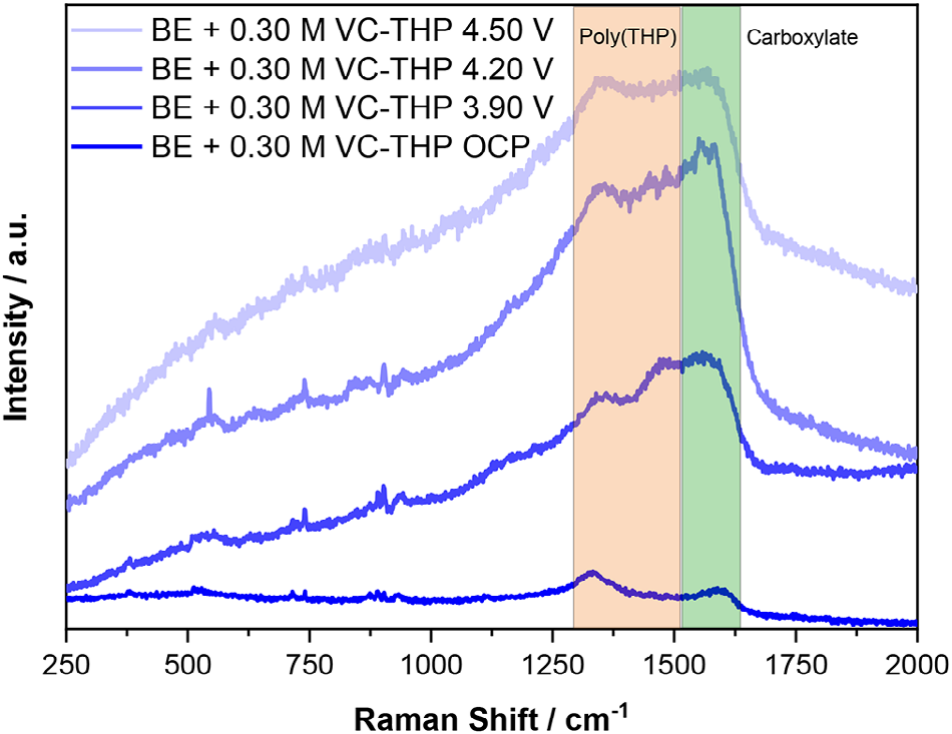
SHINER spectra recorded from the surface of a NMC811 electrode in the presence of the baseline electrolyte (1 m LiPF_6_ in EC:EMC, 3:7 by weight) + 0.30 m VC‐THP. Spectra were recorded at different electrode potentials, measured against a Li‐metal reference electrode (potentials are noted vs Li|Li^+^). Relevant regions of the spectra are highlighted by colored boxes, and the assignment of the respective bands is noted.

*Operando* SHINERS measurements were performed on the surface of NMC811 electrodes in the presence of the BE + 0.30 m VC‐THP. It should be noted that for the SHINERS measurements, a higher concentration of 0.30 m VC‐THP was employed to enable effective interphase characterization, instead of the optimized formulation of 0.05 m VC‐THP. For the experiment, the NMC811 positive electrode was paired against an AG + 20% SiO*_x_* negative electrode and a Li‐metal reference electrode. As shown in previous studies of our group, a protective CEI is not expected to form only from BE itself.^[^ ^43^ ^]^ Additional Raman measurements performed in the presence of the BE confirmed this suggestion (Figures S12 and S13, Supporting Information). In contrast, as depicted in **Figure** **8** below, additional prominent bands arise in the SHINER spectra ≈1370 and 1500 cm^−1^ in the presence of VC‐THP. Both bands can be attributed to the presence of polythiophene on the surface of the cathode, demonstrating the suggested oxidative polymerization of the thiophene motif on the positive electrode.^[^ ^43^, ^45^, ^46^ ^]^

Furthermore, these bands were observed at elevated electrode potentials of ≥3.90 V versus Li|Li^+^, agreeing with the results of the CV experiments. Besides the bands ascribed to polythiophene, another band was observed ≈1560 cm^−1^. This band can typically be attributed to the presence of carboxylates. This is in good agreement with the DFT calculations and XPS findings, which showed that the carboxylic group of the connecting bridge likely remains bond to the THP molecule (see Section S3 and Figures S7 and S8, Supporting Information). The observation of the band ≈1560 cm^−1^, as well as the finding that it arises at the same electrode potential as the polythiophene bands, supports the conclusion that the additive‐derived CEI might consist of polymerized thiophene carboxylic acid. As no other prominent bands were observed in the SHINER spectra, it can be further concluded that the thiophene‐based polymer makes up the majority of the formed interphase. While the discussed bands are highly characteristic for the assigned molecules, it has to be considered that especially the bands ≈1370 and 1560 cm^−1^ could also be attributed to the graphitic conductive agent, present in the cathode active material. To clarify the origin of the bands observed during the SHINERS experiments, additional SERS experiments were performed on the surface of a roughened Au electrode in the presence of the BE + VC‐THP. As depicted in Figure S11 (Supporting Information) the obtained SER spectra exhibit the same characteristic bands ≈1370, 1500, and 1560 cm^−1^, linking their origin exclusively to the presence of the VC‐THP additive.

### 2.5 DFT Analysis and Proposed In Situ Splitting Mechanism of the Additive

Density functional theory (DFT) calculations were performed to investigate the reduction behavior and plausible in situ splitting mechanism of the VC‐THP additive. The lowest unoccupied molecular orbital (LUMO) energy of VC‐THP was found to be significantly lower than that of EC and VC (Figure S9, Supporting Information), indicating a higher reduction propensity at the anode.

Experimentally CV measurements conducted using AG + 20% SiO_x_||Li‐metal T‐type Swagelok cells, where lithium metal served as the reference electrode revealed an additional reduction peak at 1.43 V versus Li|Li⁺ during the first cycle, which we attribute to the initial electrochemical reduction of VC‐THP (Figure S5, Supporting Information).

DFT calculations further suggest that the C─O bond connecting the VC and thiophene motifs is the weakest linkage in the molecule. Upon one‐electron reduction, this bond is predicted to cleave, yielding two fragments: a dimethyl VC radical and a thiophene carboxylate radicle. The LUMO energy of the thiophene carboxylate fragment was calculated to be lower than that of the dimethyl VC radical, making it more likely to undergo a subsequent reduction (Figure S10, Supporting Information).

**Figure 9:**
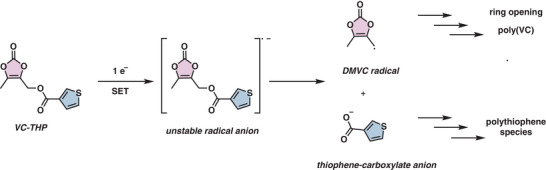
Plausible in situ splitting mechanism of the proposed bifunctional additive (VC‐THP).

Based on both theoretical and experimental data, we propose the following mechanism (**Figure** **9**): VC‐THP undergoes a one‐electron reduction at the anode, triggering C─O bond cleavage. The resulting dimethyl VC radical contributes to SEI formation through known VC polymerization pathways, consistent with the detection of (poly)‐VC species on the anode via XPS. Simultaneously, the thiophene carboxylate anion is further reduced and polymerized at the cathode, forming a polythiophene‐based CEI. This is supported by the presence of sulfur‐containing species in the XPS spectra of the cycled cathode and *operando*‐Raman data, indicating the formation of thiophene‐derived CEI components.

## 3 Conclusion

In conclusion, we successfully designed and synthesized a novel bifunctional electrolyte additive by combining two well‐established additive motifs in one molecule, each contributing distinct advantages in forming stable and conductive interphases on both the anode and cathode. This dual‐functionality enables stabilization of the SEI and the CEI at the same time, addressing a key challenge in high‐energy LIBs. Our additive demonstrated a noteworthy improvement in electrochemical performance, as confirmed through galvanostatic charge/discharge cycling experiments. Through a comprehensive set of analytical techniques—including XPS, SEM, *operando* SHINERS, and DFT calculations—we verified the additive's in situ splitting mechanism. The VC fragment preferentially contributes to SEI formation on the anode, while the carboxylate‐thiophene fragment exclusively integrates into the CEI at the cathode.

Overall, our findings establish a new design principle for electrolyte additives that simultaneously stabilize both electrode interphases, an essential requirement for improving the safety, longevity, and energy density of next‐generation LIBs and beyond cell chemistries. This work enables new strategies for developing advanced electrolyte formulations capable of addressing the increasing demands of high‐performance energy storage systems.

## 4 Experimental Section

### Additive Synthesis

5‐methyl‐2‐oxo‐1,3‐dioxol‐4‐yl)methyl thiophene‐3‐carboxylate (VC‐THP) was synthesized following a modified procedure. In a round‐bottom flask equipped with a PTFE‐coated stirring bar, the DMVC‐OH (3.8 mmol, 500 mg, 1.3 equiv.), EDC·HCl (5.76 mmol, 848.2 mg, 1.5 equiv.), the thiophene‐3‐carboxylic acid (2.9 mmol, 371.5 mg, 1.0 equiv.), DMAP (3.3 mmol, 410.8 mg, 1.14 equiv.) were dissolved in CH_2_Cl_2_ (1 mL mmol^−1^). The reaction was stirred at room temperature overnight, then poured in water (approx. 5 mL mmol^−1^). The layers were separated, then the aqueous layer was extracted once with CH_2_Cl_2_ (approx. 5 mL mmol^−1^), then the combined organic layers were washed once with distilled water (approx. 5 mL mmol^−1^), dried over MgSO_4_, and the solvent was removed *in vacuo*. The crude residue was purified by flash column chromatography on silica (n‐pentane/EtOAc mixtures), to afford the desired product. Complete characterization data of the additive is summarized in Supporting Information.

### Electrolyte Preparation

For the galvanostatic cycling experiments, different electrolyte formulations were prepared based on a baseline electrolyte (BE), consisting of 1.0 m lithium hexafluorophosphate (LiPF_6_, battery grade, E‐Lyte Innovations, Germany), dissolved in a mixture of ethylene carbonate and ethyl methyl carbonate in a ratio of 3:7 by weight (both: battery grade, E‐Lyte Innovations, Germany). To the BE VC (battery grade, E‐Lyte Innovations, Germany) and the synthesized VC‐THP were added in various concentrations, ranging from 0.05 m to 0.30 m. Note that the volume of the utilized EC:EMC mixture was adjusted to keep the concentration of LiPF_6_ in the resulting formulation at 1.0 m. All electrolyte formulations were prepared and stored in an argon‐filled glovebox (MBraun, Germany, H2O, and O2 < 1 ppm).

### Cell Preparation

Galvanostatic charge/discharge cycling of the electrolyte formulations was carried out using NMC811||AG + 20% SiO*_x_* commercial pouch‐cells (Li‐FUN Technology, Hunan, China) with an NMC811 active mass loading of 15.28 mg cm‐^2^ and a nominal capacity of 250 mAh. Before cell assembly, the pouch cells were opened and dried under reduced pressure at 90 °C for 12 h. Afterward, the cells were filled with 700 µL of electrolyte and massaged gently for ≈5 min to ensure sufficient wetting of the cell stack. The cells were vacuum‐sealed on the upper end of the gas pocket at 165 °C for 5 s using a pouch‐cell sealer (GN‐HS350V, Gelon LIB Co., Shangdong, China). The sealing process was carried out at 15% of the ambient pressure. For degassing of the cells after formation, the cells were cut open and sealed directly at the cell stack under the aforementioned conditions. Preparation and degassing of the cells were carried out in a dry‐room (dew point <−50 °C).

### Cyclic Voltammetry Studies

Potentiodynamic characterization of the considered electrolyte formulations via cyclic voltammetry (CV) was carried out using Swagelok T‐type cells, allowing the use of a three‐electrode configuration for precise determination of the electrode potential. For cell preparation, the inside of the Swagelok cells was covered with Mylar Foil (PET) to ensure electronic insulation. WE and CE were separated by a 13 mm Whatman GF/D glass fiber separator, soaked with 120 µL of electrolyte. The RE was separated by an additional 10 mm Whatman GF/D glass fiber separator, soaked with 80 µL of electrolyte. The electrodes used for CV investigation were taken from the pouch cells. This was achieved by opening the cells, removing one side of the coating with THF (VWR, USA), and punching 12 mm diameter electrodes from the AG + 20% SiO*_x_* and NMC811 electrode sheets. Before usage, the electrodes were dried at 90 °C under reduced pressure overnight. Cell preparation was performed in an argon‐filled glovebox.

### Electrochemical Investigation

Galvanostatic charge/discharge cycling of the cells was carried out using a Maccor 4000 series battery tester in a voltage range from 4.50 to 2.80 V. Before cycling, the cells were pre‐polarized from open circuit voltage to 1.50 V for 12 h. For cell formation two charge/discharge cycles at C/10 with constant current/constant voltage (CCCV) steps were performed, followed by the degassing of the cells. Afterward, the cells were connected again to the battery tester and rested for 12 h before continuous CCCV charge/discharge cycling at 1 C. Note that the applied C‐rate was calculated based on the nominal capacity of the pouch cells of 250 mAh. Galvanostatic charge/discharge cycling of the cells was performed in temperature‐controlled chambers (BINDER, Germany) at 20 °C. To apply a constant pressure of ≈2 bar on the cell stack during charge/discharge cycling, the prepared cells were attached to specially designed cell holders.^[^ ^48^ ^]^

Potentiodynamic investigations of the prepared electrolyte formulations were conducted via CV measurements, carried out on an Autolab Battery Tester (Metrohm, Switzerland) controlled by NOVA 2.1 (Metrohm, Switzerland) with a sweep rate of 150 µV s^−1^. Cells with an NMC811||AG + 20% SiO*_x_* chemistry were evaluated between 2.80 V versus Li|Li^+^ and 4.50 V versus Li|Li^+^, and cells with an AG + 20% SiO*_x_* ||Li‐metal cell chemistry were investigated between 2.00 V versus Li|Li^+^ and 0.05 V versus Li|Li^+^. Li‐metal was used as reference electrode for all CV measurements. CV experiments were performed for the BE and the BE with an additional 0.30 m VC‐THP. Before the measurement, the cells were rested for 10 h.

### X‐Ray Photoelectron Spectroscopy (XPS) Measurements

XPS measurements were performed on Thermo Fisher Scientific K‐Alpha instruments. The instrument parameters are summarized in Table S1 (Supporting Information). All spectra were referenced to C1s at 284.8 eV. The spectra were analyzed by the use of Avantage 5.9925 (Thermo Fisher Scientific). Data visualization was done with Microsoft Excel. Electrode samples for XPS investigations were taken from NMC811||AG + 20% SiO*_x_* multilayer pouch cells, after performing two formation cycles in the presence of the BE, the BE + 0.15 m VC, the BE + 0.05 m THP, and the BE + 0.05 m VC‐THP. For sample preparation, the electrochemically aged pouch cells were transferred to an argon‐filled glovebox, opened, and electrode samples cut. Subsequently, the samples were washed with 1000 µL EMC to remove electrolyte residues and dried under reduced atmosphere. To avoid contamination the samples were transferred to the XPS using an air‐tight vessel.

### Operando Raman Measurements

The sample for SHINERS experiments were prepared according to standard protocol reported in the previous works.^[^ ^43^ ^]^ The Raman measurements were conducted using a confocal Raman microscope (LabRam Evolution HR, Horiba Scientific), equipped with an air‐cooled CCD detector, a 600 line mm^−1^ grating, and a 50 x long‐working distance objective (9.2 mm, numerical aperture 0.5, Zeiss, Germany). The samples were excited with a red laser (633 nm, 30 s, 4 x). Laser power was reduced to 1.05 mW by a 10% filter. Before every measurement, the system was calibrated to the peak of crystalline silicon at 520.70 cm^−1^. Operation of the Raman system, recording, and evaluation of the spectra were performed by using the LabSpec6.7.1.10 software (Horiba Scientific).

For the Raman measurements, potentiodynamic charging of the optical cells was performed, using an Autolab potentiostat/galvanostat PGSTAT204 (Metrohm, Switzerland) controlled by the NOVA 2.1 software (Metrohm, Switzerland). Raman measurements were performed at the OCP and electrode potentials of 3.90 V versus Li|Li^+^, 4.20 V versus Li|Li^+^, and 4.50 V versus Li|Li^+^. During Raman measurements, a constant voltage was applied to the electrode to avoid a voltage drop.

### Scanning Electron Microscopy (SEM) Measurements

Surface morphology investigations were performed via SEM, using an Auriga electron microscope (Carl Zeiss Microscopy, Germany) with an acceleration voltage of 3 kV. Electrode samples were taken from NMC811||AG + 20% SiO*_x_* multilayer pouch cells, after performing two formation cycles in the presence of the BE, the BE + 0.15 m VC, and the BE + 0.05 m VC‐THP. For sample preparation, the electrochemically aged pouch cells were transferred to an argon‐filled glovebox, opened, and electrode samples cut. Subsequently, the samples were washed with 1000 µL EMC to remove electrolyte residues and dried under reduced atmosphere. To avoid contamination the samples were transferred to the SEM using an air‐tight vessel.

### Density Functional Theory (DFT) Calculations

DFT calculations were performed to gather additional information on the additive molecule. The calculations were performed using the Gausian16 package.^[^ ^49^ ^]^ Molecule geometries were optimized using the B3LYP DFT and 6‐311++G(3df, 2p) basis set. The SMD implicit solvation model was employed to mimic the effect of the surrounding electrolyte. For the solvation model the parameters of acetone were chosen, which show a dielectric constant similar to carbonate‐based electrolytes.^[^ ^50^ ^]^

## Conflict of Interest

The authors declare no conflict of interest.

## Supporting information



Supporting Information

## Data Availability

The data that support the findings of this study are available in the supplementary material of this article.
