# Supplementary material for: Bifunctional Electrolyte Additive Enabling Simultaneous Interphase Formation on Both Electrodes in High‐Energy Lithium‐Ion Batteries
Source: Small. 2025 Sep 1;21(42):e05772. doi: 10.1002/smll.202505772 (PMC12548004; doi:10.1002/smll.202505772)
Supplement: Supplementary file 1 — Supporting Information [file SMLL-21-e05772-s001.docx]

Supporting Information

**Bifunctional Electrolyte Additive Enabling Simultaneous Interphase Formation on Both Electrodes in High-Energy Lithium-Ion Batteries**

Ankita Das^1,3,§^, Felix Pfeiffer^2,§^, Anindityo Arifiadi^3,4^, Matthias Weiling^2^, Verena Küpers^4^, Masoud Baghernejad^2^*, Martin Winter^2,4^, Frank Glorius^1,3^*

A. Das, Prof. F. Glorius*

^1^University of Münster, Institute of Organic Chemistry

Corrensstr. 36, 48149 Münster, Germany

*E-mail: glorius@uni-muenster.de

F. Pfeiffer, M. Weiling, M. Winter, M. Baghernejad*

^2^Helmholtz Institute Münster, IMD-4, Forschungszentrum Jülich GmbH

Corrensstr. 46, 48149 Münster, Germany

*E-mail: b.masoud@fz-juelich.de

A. Das, A. Arifiadi, F. Glorius

^3^University of Münster, International Graduate School for Battery Chemistry, Characterization, Analysis, Recycling and Application (BACCARA)

Corrensstr. 40, 48149 Münster, Germany

A. Arifiadi, V. Küpers, M. Winter

^4^University of Münster, MEET Battery Research Center

Corrensstr. 46, 48149 Münster, Germany

^§^*A. Das* and *F. Pfeiffer* contributed equally to this work.

**Contents**

[**1.** **Synthesis** S1](#_Toc198037398)

[**1.1** **General Information** S1](#_Toc198037399)

[**1.2 Synthesis of the bifunctional additive** S1](#_Toc198037400)

[**1.3 NMR characterization data of the bifunctional additive** S2](#_Toc198037401)

[**2.** **Electrochemical Investigations** S4](#_Toc198037402)

[**3.** **Potentiostatic Investigation** S7](#_Toc198037403)

[**4.** **DFT calculations** S8](#_Toc198037404)

[**5.** **Surface enhanced Raman spectroscopy** S9](#_Toc198037405)

[**6.** **XPS Measurements** S11](#_Toc198037406)

# **Synthesis**

## **General Information**

All reactions were carried out in oven-dried glassware with oven-dried Teflon-coated magnetic stir bars. Dry solvents were either taken from a solvent purification system (HPLC grade, dried over activated alumina columns) or purchased from Acros Organics, Sigma-Aldrich or Carl Roth (stored over activated molecular sieves). All reagents were obtained from ABCR, Acros Organics, Alfa Aesar, Carbolution Chemicals, Carl Roth, Chempur Combi-Blocks, Fisher Scientific, Fluorochem, Merck, Sigma-Aldrich, TCI Europe or VWR and utilized as received.

**1H- and 13C-NMR spectra** were recorded on a Bruker AV 400 at room temperature. Chemical shifts (δ) were given in ppm. The residual solvent signals were used as references and the chemical shifts converted to the TMS scale (MeOD: δH = 4.87 ppm, δC = 49.00 ppm; CD_2_Cl_2_: δH = 5.32 ppm, δC = 54.00 ppm; D_2_O: δH = 4.79 ppm,). All the NMRs were processed using Mestrenova 14 applying standard phase and baseline corrections. Coupling constants (*J*) are quoted in Hz.

**High resolution mass spectra (HRMS)** were recorded on a Thermo Scientific Exploris 120 Electrospray Orbitrap in electrospray ionization mode (ESI). ESI spectra show relative abundance after normalization against maximum signal intensity level (NL) in dependence of m/z.

## **1.2 Synthesis of the bifunctional additive**

**Scheme S1.** Synthesis of (5-methyl-2-oxo-1,3-dioxol-4-yl) methyl thiophene-3-carboxylate.

In a round-bottom flask equipped with a PTFE-coated stirring bar, DMVC-OH (3.8 mmol, 500 mg, 1.3 equiv.), EDC·HCl (5.76 mmol, 848.2 mg, 1.5 equiv.), the thiophene-3-carboxylic acid (2.9 mmol, 371.5 mg, 1.0 equiv.), DMAP (3.3 mmol, 410.8 mg, 1.14 equiv.) were dissolved in CH_2_Cl_2_ (1 mL mmol^-1^). The reaction was stirred at room temperature overnight before being poured into water (approx. 5 mL/mmol). The organic and aqueous layers were separated, then the aqueous layer was extracted once with CH_2_Cl_2_ (approx. 5 mL/mmol). The combined organic layers were then washed once with distilled water (approx. 5 mL/mmol), dried over MgSO_4_ and the solvent was removed under reduced pressure. The crude residue was purified by flash column chromatography on silica (*n*-pentane/EtOAc mixtures), yielding the desired product as a colorless liquid.

**^1^H NMR** (400 MHz, CDCl_3_) δ 8.15 (dd, *J* = 3.1, 1.2 Hz, 1H), 7.51 (dd, *J* = 5.1, 1.2 Hz, 1H), 7.32 (dd, *J* = 5.1, 3.0 Hz, 1H), 5.02 (s, 2H), 2.22 (s, 3H).

**^13^C NMR** (101 MHz, CDCl_3_) δ 162.1, 152.2, 140.3, 133.8, 133.7, 132.4, 127.9, 126.5, 54.0, 9.5.

**HRMS (ESI+):** m/z calculated for C_10_H_8_O_5_SNa^+^ [M+Na]^+^: 262.9985; found: 263.0844.

## **1.3 NMR characterization data of the bifunctional additive**

*^1^H spectrum (400 MHz, CDCl_3_)*

**

*^13^C spectrum (100 MHz, CDCl_3_)*

# **Electrochemical Investigations**


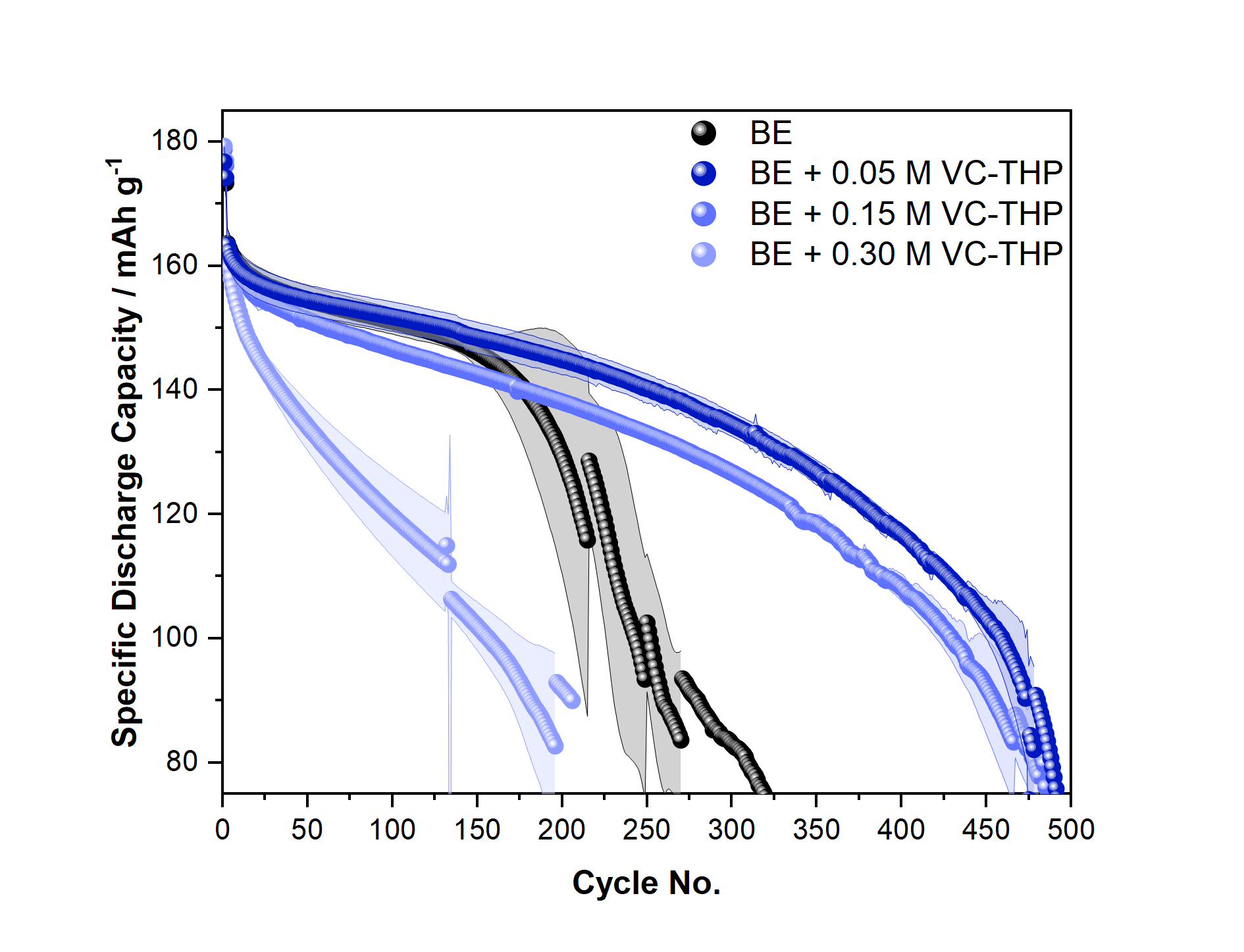


**Figure S1**. Specific discharge capacities obtained during the galvanostatic cycling experiments of NMC811||AG + 20 % SiO_x_ pouch cells. The cells were cycled in the presence of the baseline electrolyte (BE, 1 M LiPF_6_ in EC:EMC, 3:7 by weight; **black**) and the BE + different VC-THP concentrations (**blue**).


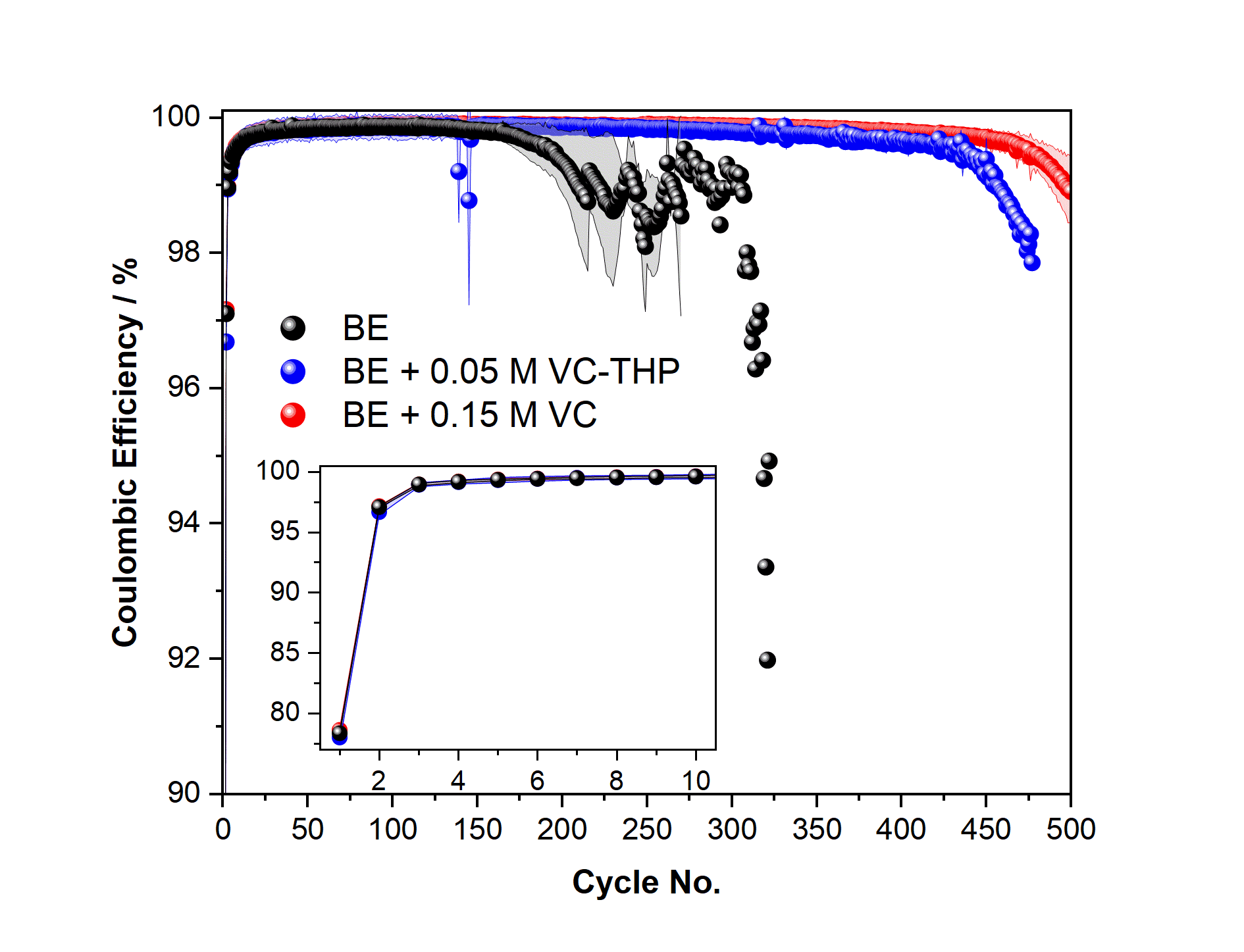


**Figure S2**. Coulombic efficiencies obtained during the galvanostatic cycling experiments of NMC811||AG + 20 % SiO_x_ pouch cells. The cells were cycled in the presence of the baseline electrolyte (BE, 1 M LiPF_6_ in EC:EMC, 3:7 by weight; **black**), the BE + 0.05 M VC-THP concentrations (**blue**), and the BE + 0.15 M VC (**red**).


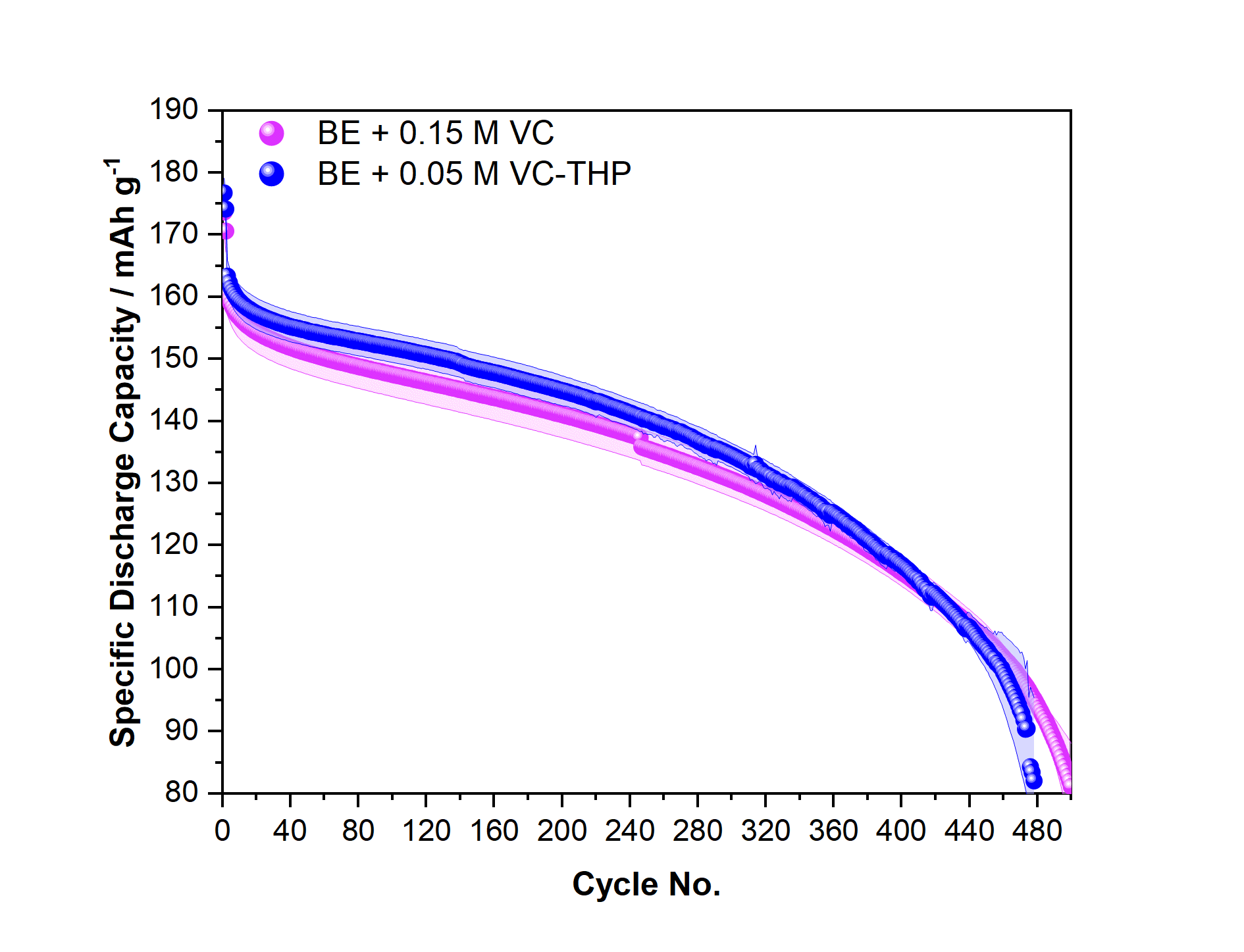


**Figure S3**. Specific discharge capacities obtained during the galvanostatic cycling experiments of NMC811||AG + 20 % SiO_x_ pouch cells. The cells were cycled in the presence of the baseline electrolyte (BE, 1 M LiPF_6_ in EC:EMC, 3:7 by weight) + 0.15 M VC (**purple**) and the BE + 0.05 M VC-THP (**blue**).


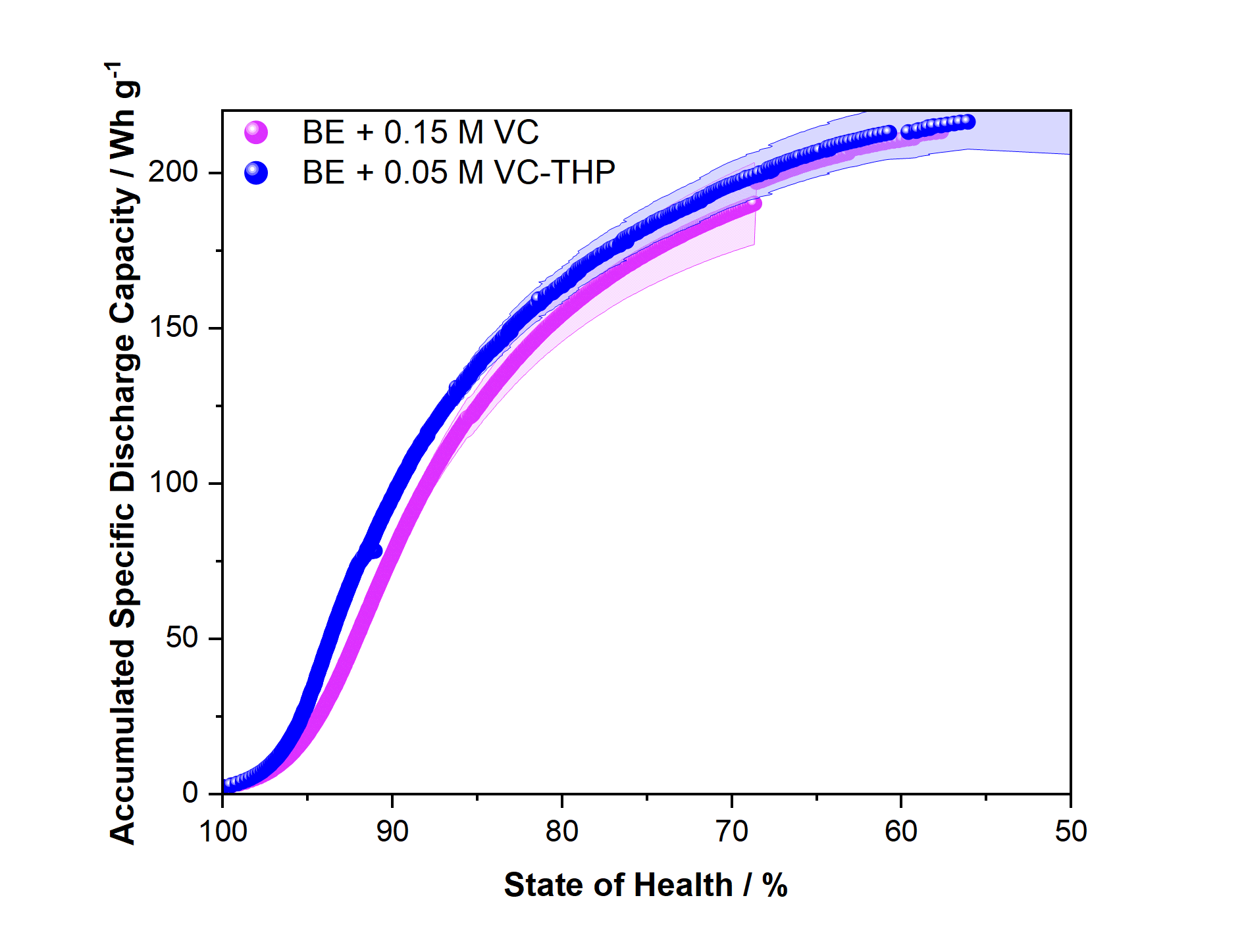


**Figure S4**. Accumulated specific discharge energies obtained from NMC811||AG + 20 % SiO_x_ pouch cells during galvanostatic cycling. The cells were cycled in the presence of the baseline electrolyte (BE, 1 M LiPF_6_ in EC:EMC, 3:7 by weight) + 0.15 M VC (**purple**) and the BE + 0.05 M VC-THP (**blue**).


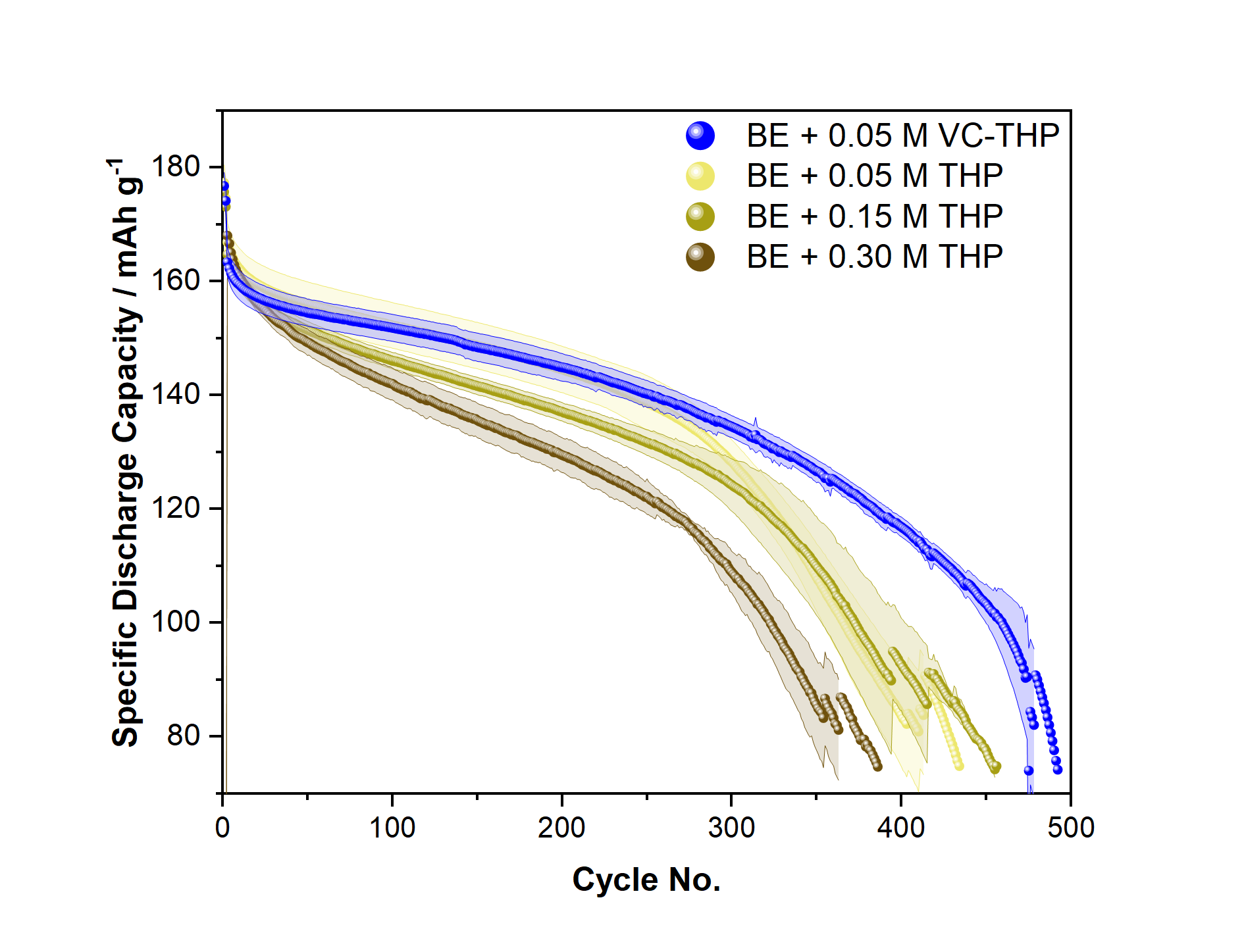


**Figure S5**. Specific discharge capacities obtained during the galvanostatic cycling experiments of NMC811||AG + 20 % SiO_x_ pouch cells. The cells were cycled in the presence of the baseline electrolyte (BE, 1 M LiPF_6_ in EC:EMC, 3:7 by weight) + 0.05 M VC-THP (**blue**) and the BE + different thiophene concentrations (0.05 M, 0.15 M, and 0.30 M; **yellow**)

# **Potentiostatic Investigation**


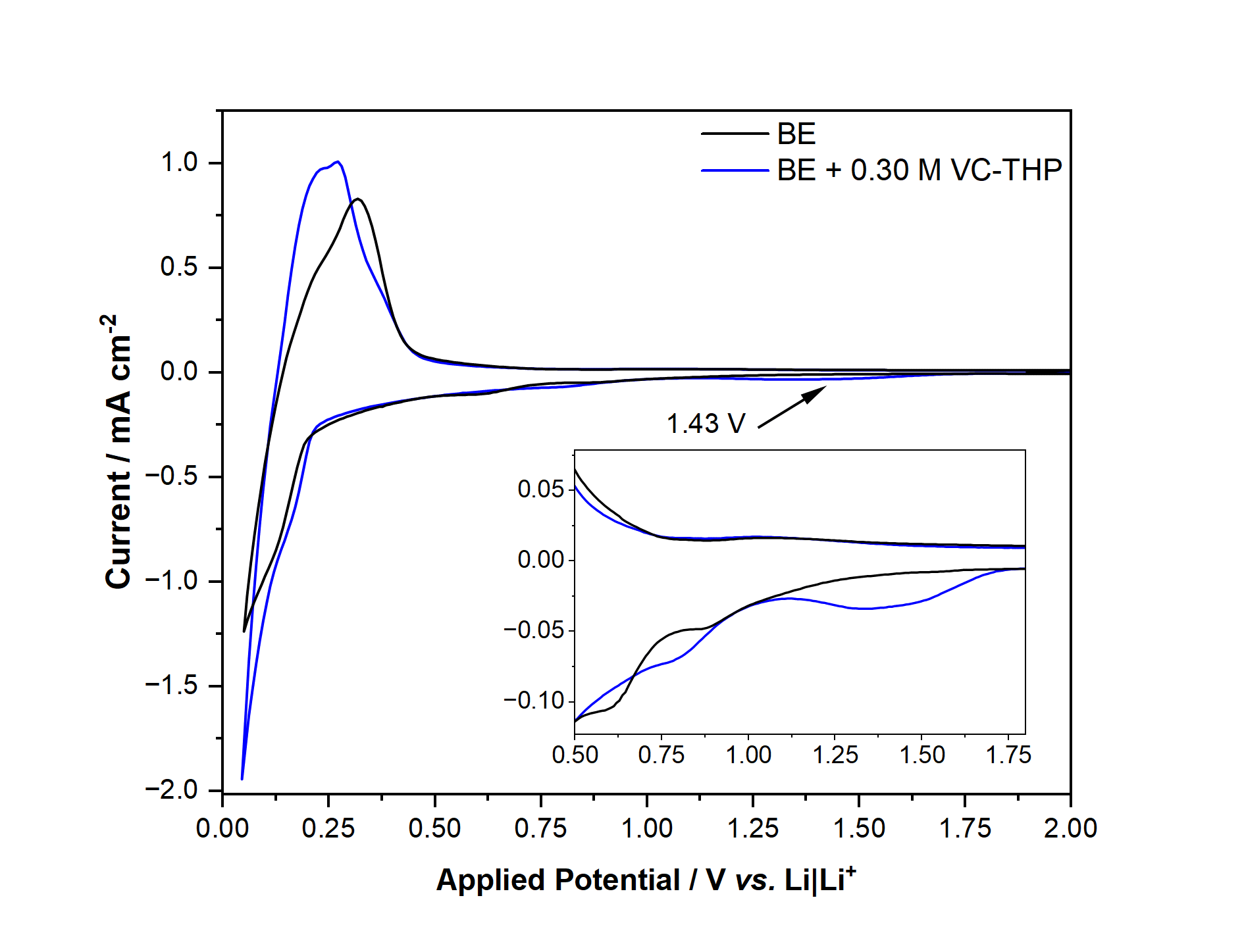


**Figure S6**. Cyclic voltammograms of AG + 20 % SiO_x_||Li-metal T-type Swagelok cells with Li-metal reference electrodes. Cyclic voltammetry was performed in the presence of the baseline electrolyte (BE, 1 M LiPF_6_ in EC:EMC, 3:7 by weight; **black**) and the BE + 0.05 M VC-THP (**blue**). Note that for both electrolyte formulations only the first cycle is shown. Relevant features are highlighted by an arrow and the respective applied potential is noted.

# **DFT calculations**

DFT calculations were performed to investigate the reductive decomposition of the VC-THP additive. Bond order analysis revealed that the C−O bond between the carboxyl group and the VC motif had the lowest value of 0.84 (**Figure S7**). Upon reduction, this bond is likely to break first, resulting in the separation of the VC and the THP motif. Additionally, the DFT results indicate that the carboxyl group remains attached to the THP motif. These findings align with the observations from electrochemical experiments.


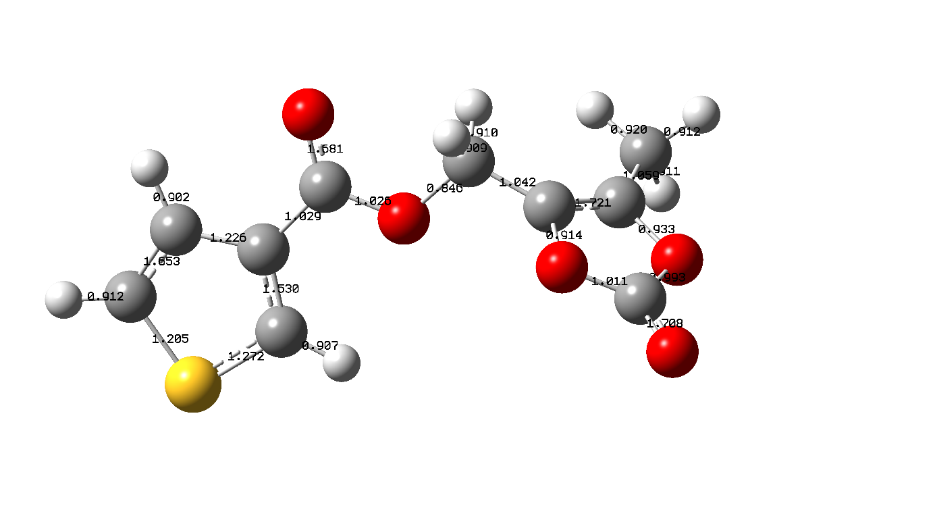


**Figure S7**. Bond orders calculated for the VC-THP molecule via DFT.


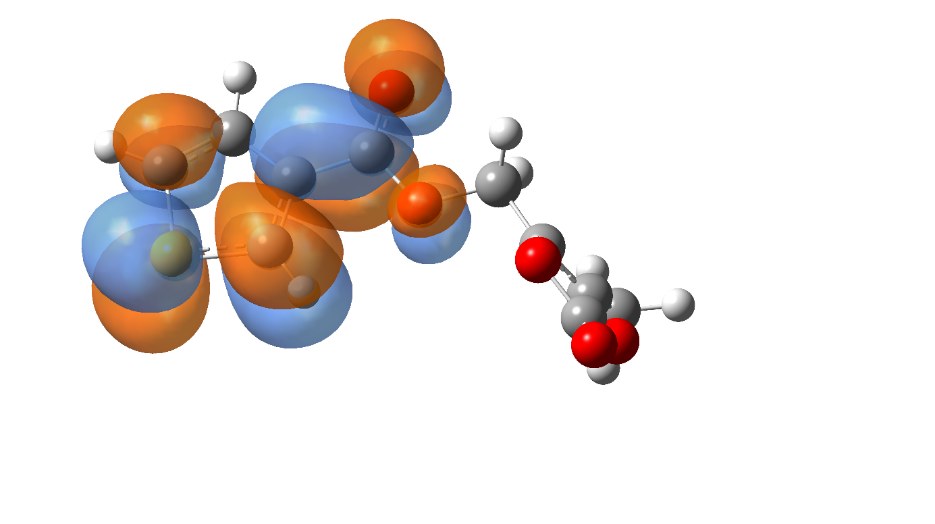


**Figure S8**. LUMOs calculated for the VC-THP molecule via DFT.


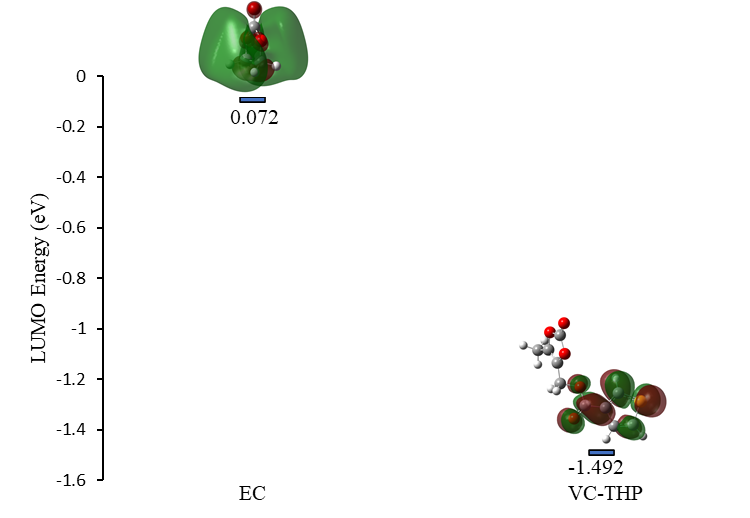


**Figure S9.** LUMO energy levels of the EC and VC-THP.


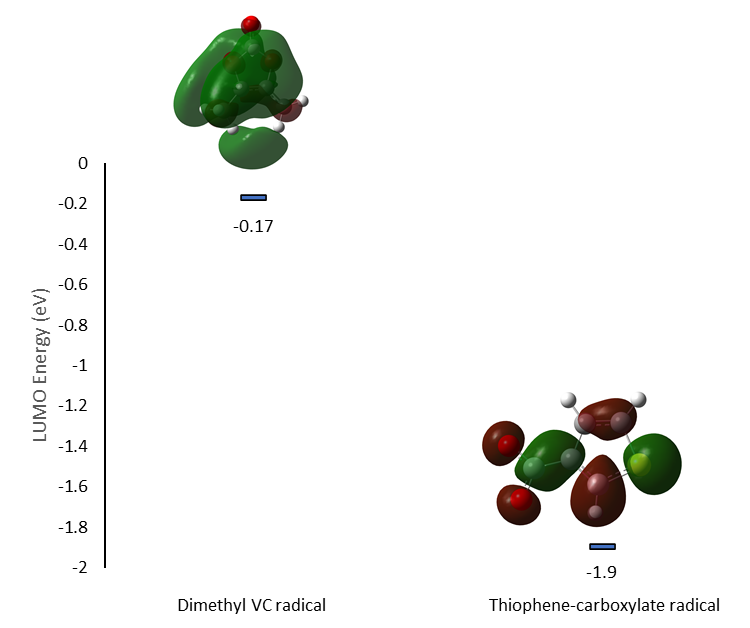


**Figure S10**. LUMO energy levels of the neutrally charged dimethyl VC radical and thiophene-carboxylate radical cleavage structures, which are possible products from the decomposition of VC-THP.

# **Surface enhanced Raman spectroscopy**

**Figure S11**. SHINER spectra of the surface of a NMC811 electrode in the presence of the baseline electrolyte (BE, 1 M LiPF_6_ in EC:EMC, 3:7 by weight). Depicted are the spectra recorded at the OCP (grey) and the cut-off potential of 4.50 V. All given potentials given in the Li|Li^+^ scale, referenced against a Li-metal reference electrode.

For the SERS measurements, a gold foil (99.995%, ChemPUR, Germany) was first annealed using a gas burner until the electrode was glowing red. This state was held for at least 1 min. Afterward, the electrode was electrochemically roughened in a 0.10 M KCl (>99.50%, AppliChem, Germany) bath. Therefore, the gold electrode was cycled between −0.30 and +0.90 V *vs.* Pt with a sweep rate of 200 µV s^−1^. The potential was held at the ends of the sweep for 2 and 9 s, respectively. Platinum was used as CE and RE. Afterward, the electrode was cleaned with Milli-Q water and dried in an N_2_ stream.

For the SERS measurements, a similar set-up as the *operando* Raman measurements (see experimental section) was used, featuring the roughened Au-electrode as WE at the bottom, topped by the separator and an NMC811 counter electrode with circular cut-outs and a Li-metal reference electrode.

**Figure S12**. SER spectra of the surface of a roughened Au electrode in the presence of the baseline electrolyte (BE, 1 M LiPF_6_ in EC:EMC, 3:7 by weight). Depicted are the spectra recorded at the OCP (grey) and the cut-off potential of 4.50 V. All given potentials given in the Li|Li^+^ scale, referenced against a Li-metal reference electrode.

**Figure S13**. SER spectra of the surface of a roughened Au electrode in the presence of the baseline electrolyte (BE, 1 M LiPF_6_ in EC:EMC, 3:7 by weight) + 0.30 M VC-THP. Depicted are the spectra recorded at the OCP (grey) and the cut-off potential of 4.50 V. All given potentials given in the Li|Li^+^ scale, referenced against a Li-metal reference electrode. Prominent bands observed in the spectra are highlighted by arrows and the respective Raman shift is noted.

# **XPS Measurements**

**Table S1**. XPS measurement parameters.

| **XPS** | **X-ray source** | **Spot and detection** | **Charge compensation** | **Pass Energy (eV)** | **Dwell time**  **(ms)** | **Step size (eV)** | **No. of Scans** |
| --- | --- | --- | --- | --- | --- | --- | --- |
| Survey | Al Kα, 12 kV filament | 100 µm (72W max.). 60° x-ray incidence angle, 0° take-off angle (rel. to surface normal) | Metallic clips on thin film connected to grounded sample stage. | 200 | 100 | 0.9 | 2 |
| Li1s |  |  |  | 50 | 50 | 0.05 | 5 |
| N1s |  |  |  | 50 | 50 | 0.05 | 5 |
| C1s |  |  |  | 50 | 50 | 0.05 | 5 |
| O1s |  |  |  | 50 | 50 | 0.05 | 5 |
| P2p |  |  |  | 50 | 50 | 0.05 | 5 |
| S2p |  |  |  | 50 | 50 | 0.05 | 5 |
| F1s |  |  |  | 50 | 50 | 0.05 | 5 |

**Table S2**. Normalized proportion of chemical bonds of XPS S 2p spectra of NMC811 cathodes precycled with a) VC-THP, b) thiophene (THP), c) VC, d) baseline electrolyte (BE, 1 M LiPF_6_ in EC:EMC, 3:7 by weight).

| **S 2p** | **168.3**  **SO_4_^2-^** | **164.1**  **Poly-THP** |
| --- | --- | --- |
| **a) VC-THP** | 81.8 | 18.2 |
| **b) THP** | 16.0 | 84.0 |
| **c) VC** | 0.0 | 0.0 |
| **d) BE** | 0.0 | 0.0 |

**Table S3**. Normalized proportion of chemical bonds of XPS O 1s spectra of NMC811 cathodes precycled with a) VC-THP, b) thiophene (THP), c) VC, d) baseline electrolyte (BE, 1 M LiPF_6_ in EC:EMC, 3:7 by weight).

| **O 1s** | **533.5**  **C−O** | **531.6**  **C=O** | **528.7**  **M−O** |
| --- | --- | --- | --- |
| **a) VC-THP** | 25.7 | 61.6 | 12.7 |
| **b) THP** | 29.3 | 53.1 | 17.5 |
| **c) VC** | 24.8 | 60.0 | 15.2 |
| **d) BE** | 22.9 | 60.9 | 16.2 |

**Table S4**. Normalized proportion of chemical bonds of XPS F 1s spectra of NMC811 cathodes precycled with a) VC-THP, b) thiophene (THP), c) VC, d) baseline electrolyte (BE, 1 M LiPF_6_ in EC:EMC, 3:7 by weight).

| **F 1s** | **687.4**  **P−F/ C−F** | **684.9**  **Li−F** |
| --- | --- | --- |
| **a) VC-THP** | 76.6 | 23.4 |
| **b) THP** | 93.5 | 6.5 |
| **c) VC** | 91.4 | 8.6 |
| **d) BE** | 80.0 | 20.0 |

**Table S5**. Normalized proportion of chemical bonds of XPS C 1s spectra of Si-C anodes precycled with a) VC-THP, b) thiophene (THP), c) VC, d) baseline electrolyte (BE, 1 M LiPF_6_ in EC:EMC, 3:7 by weight).

| **C 1s** | **291.3**  **Poly-VC** | **289.9**  **CO_3_^2-^** | **288.4**  **C=O** | **286.7**  **C−O** | **284.8**  **C−C** | **282.8**  **LiC_6_** |
| --- | --- | --- | --- | --- | --- | --- |
| **a) VC-THP** | 1.5 | 5.7 | 3.9 | 14.5 | 68.8 | 5.5 |
| **b) THP** | 0 | 2.1 | 2.4 | 6.1 | 86.6 | 2.8 |
| **c) VC** | 0.9 | 5.9 | 5.3 | 18.4 | 63.6 | 5.9 |
| **d) BE** | 0 | 8.4 | 3.8 | 15.5 | 63.9 | 8.4 |

**Table S6**. Normalized proportion of chemical bonds of XPS O 1s spectra of Si-C anodes precycled with a) VC-THP, b) thiophene (THP), c) VC, d) baseline electrolyte (BE, 1 M LiPF_6_ in EC:EMC, 3:7 by weight).

| **O 1s** | **533.7**  **O−C−O** | **531.8**  **C=O** | **528.6**  **M−O** |
| --- | --- | --- | --- |
| **a) VC-THP** | 19.6 | 80.4 | 0 |
| **b) THP** | 10.4 | 81.5 | 8.1 |
| **c) VC** | 19.3 | 80.7 | 0 |
| **d) BE** | 18.7 | 81.3 | 0 |

**Table S7**. Normalized proportion of chemical bonds of XPS F 1s spectra of Si-C anodes precycled with a) VC-THP, b) thiophene (THP), c) VC, d) baseline electrolyte (BE, 1 M LiPF_6_ in EC:EMC, 3:7 by weight).

| **F 1s** | **687.2**  **P−F/ C−F** | **684.9**  **Li−F** |
| --- | --- | --- |
| **a) VC-THP** | 67.8 | 32.2 |
| **b) THP** | 69.2 | 30.8 |
| **c) VC** | 83.8 | 16.2 |
| **d) BE** | 63.2 | 36.8 |
